# Spatial domain analysis predicts risk of colorectal cancer recurrence and infers associated tumor microenvironment networks

**DOI:** 10.1038/s41467-020-17083-x

**Published:** 2020-07-14

**Authors:** Shikhar Uttam, Andrew M. Stern, Christopher J. Sevinsky, Samantha Furman, Filippo Pullara, Daniel Spagnolo, Luong Nguyen, Albert Gough, Fiona Ginty, D. Lansing Taylor, S. Chakra Chennubhotla

**Affiliations:** 1 Department of Computational and Systems Biology, University of Pittsburgh, Pittsburgh, PA 15260 USA; 2 University of Pittsburgh Drug Discovery Institute, University of Pittsburgh, Pittsburgh, PA 15261 USA; 3 Biology and Applied Physics, GE Global Research Center, Niskayuna, NY 12309 USA

**Keywords:** Computational biology and bioinformatics, Systems biology, Biomarkers

## Abstract

An unmet clinical need in solid tumor cancers is the ability to harness the intrinsic spatial information in primary tumors that can be exploited to optimize prognostics, diagnostics and therapeutic strategies for precision medicine. Here, we develop a transformational spatial analytics computational and systems biology platform (SpAn) that predicts clinical outcomes and captures emergent spatial biology that can potentially inform therapeutic strategies. We apply SpAn to primary tumor tissue samples from a cohort of 432 chemo-naïve colorectal cancer (CRC) patients iteratively labeled with a highly multiplexed (hyperplexed) panel of 55 fluorescently tagged antibodies. We show that SpAn predicts the 5-year risk of CRC recurrence with a mean AUROC of 88.5% (SE of 0.1%), significantly better than current state-of-the-art methods. Additionally, SpAn infers the emergent network biology of tumor microenvironment spatial domains revealing a spatially-mediated role of CRC consensus molecular subtype features with the potential to inform precision medicine.

## Abstract (web summary)

Spatial information in the tumour microenvironment may be exploited to optimise diagnosis, prognosis and therapy. Here, the authors develop a spatial analytics computational and systems pathology platform (SpAn) based on highly multiplexed antibody imaging on colorectal cancer samples to infer emergent network biology and predict 5-year risk of recurrence.

## Introduction

Colorectal Cancer (CRC) is the fourth most common type of cancer and the second leading cause of cancer-related deaths worldwide^1^. This multi-factorial disease like other carcinomas, develops and progresses through the selection of epithelial clones with the potential to confer malignant phenotypes in the context of a reciprocally coevolving tumor microenvironment (TME) comprising immune and stromal cells^2,3^. CRC patients are staged using the well-established tumor-node-metastases (TNM) classification^4,5^. However, there is significant variability in patient outcomes within each stage. For example, CRC will recur in up to 30% of Stage II patients despite complete tumor resection, no residual tumor burden and no signs of metastasis^6^. In contrast, more advanced CRC has been known to show stability or indeed even to spontaneously regress^6,7^.

The intrinsic plasticity of the TME underlying this variability in outcome is controlled by complex network biology emerging from the spatial organization of diverse cell types within the TME and their heterogeneous states of activation^8–10^. The important role of the TME in CRC progression and recurrence has recently been highlighted by the identification of four consensus molecular subtypes (CMS)^11,12^, functional studies defining the critical role of stromal cells in determining overall survival^13^, and the development of Immunoscore®^14^ which quantifies tumor-infiltrating T lymphocytes in different regions of the tumor and associates their infiltration with CRC recurrence^14,15^. However the TME can be further harnessed to significantly improve CRC prognosis through the identification of biomarkers mechanistically linked to disease progression and the development of novel therapeutic strategies.

Deeper understanding of the TME may arise from imaging methods capable of labeling >7 cellular and tissue components in the same sample (hyperplexed^16^ (HxIF) fluorescence and other imaging modalities)^16–20^. To fully extract the intrinsic information within each primary tumor we have developed a spatial analytics computational and systems pathology platform (SpAn) applicable to all solid tumors to analyze the spatial relationships throughout TME signaling networks. SpAn constructs a computationally unbiased and clinical outcome-guided statistical model enriched for a subset of TME signaling networks that are naturally selected as dependencies of the corresponding malignant phenotype. Traditionally, advances in experimental and systems biology have been made by identifying associations between differential biomarkers expressions/correlations, or clusters in T-SNE plots, with particular outcome-specific malignant phenotypes, such as cancer progression or recurrence phenotypes. Instead of using this association-driven paradigm, SpAn introduces an outcome-driven approach to predict 5-year risk of CRC recurrence in patients with resected primary tumor that also enables inference of recurrence-specific network biology.

## Results

### Hyperplexed immunofluorescence imaging of tissue microarrays

The acquired data were generated using GE Cell DIVE^TM^, also known as MultiOmyx^19^ (GE Healthcare, Issaquah, WA), hyperplexed^16^ immunofluorescence (HxIF) imaging and image processing workflow instrument. Cell DIVE^TM^ can perform hyperplexed imaging of greater than 60 biomarkers via sequentially multiplexed imaging of 2–3 biomarkers plus DAPI nuclear counterstain through iterative cycles of label–image–dye inactivation visualized in Supplementary Fig. 1^19^ (see the “Methods” section for more details). Extensive validation of this approach has demonstrated that a majority of epitopes tested are extremely robust to the dye-inactivation process. The biological integrity of the samples was preserved for at least 50 iterative cycles^19^.

**Fig. 1:**
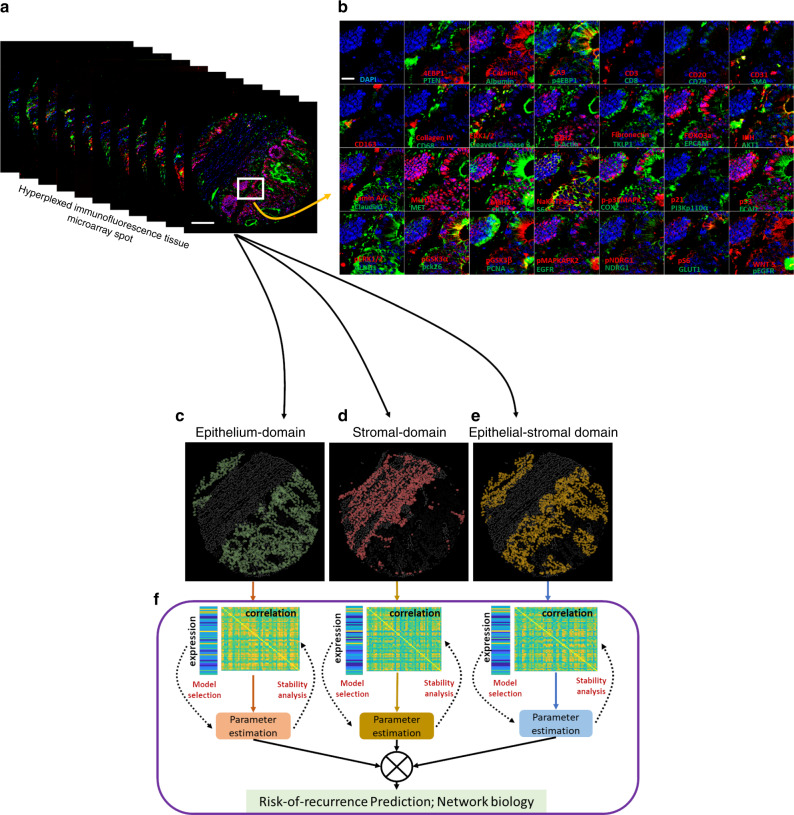
Hyperplexed immunofluorescence imaging based spatial analytics (SpAn) platform. **a** Hyperplexed image stack of a TMA spot generated by iteratively multiplexed (Fig. S2) HxIF imaging using the Cell DIVE platform^19^. Scale bar: 100 µm. **b** Close-up view of a TMA region in (**a**) outlined in white (~110 µm by 110 µm), labeled with 55 biomarkers (plus DAPI nuclear counterstain) that include epithelial, immune and stromal cell lineage, subcellular compartments, oncogenes, tumor suppressors, and post-translational protein modifications described in detail in Supplementary Table 1. Hyperplexed imaging is implemented via iterative label–image–dye-inactivation immunofluorescence cycle (see Methods and Fig. S2). Scale bar: 20 µm. **c**–**e** Dissection of the TMA spot into three spatial domains (epithelial, stromal, and epithelial-stromal domains) identified and segmented using structural biomarkers (see Methods and Fig. S3). **f** For each of the three spatial domains both expressions (scale range: 1 through 12 on a log2 scale) of the 55 biomarkers and their Kendall rank-correlations (scale range: −1 through +1) both within and across the cells together defined the domain-specific features. L1-norm based penalized Cox regression was used for model selection, while L2 penalty was used for final model parameter (coefficients) estimation. The stability of the model was tested at the 90% concordance level, and the parameters were reevaluated for final construction of the SpAn spatial model.

In this study we use 55 biomarkers, which include markers for epithelial, immune, and stromal cell lineage, along with those in categories, which include (1) biomarkers sampling the network biology of signaling pathways, (2) biomarkers associated with extracellular transport and metabolism, (3) biomarkers associated with tumor suppressive potential, (4) biomarkers associated with oncogenic potential, (5) biomarkers associated with cell–cell adhesion, cellular and stromal structure, (6) biomarkers associated with post-translational modifications (PTM), and (7) biomarkers associated with cell types and their states. They are detailed in Supplementary Fig. 2, with additional details in Supplementary Table 1. Figure 1a shows the HxIF image stack of a 5-µm thick and 0.6-mm wide tissue microarray^21^ (TMA) spot from resected primary tumor of a Stage II CRC patient labeled with the 55 biomarkers plus DAPI. Figure 1b highlights a sub-region of this patient TMA spot enabling optimal visualization of the 55 HxIF biomarker images resulting from the iterative label–image–chemical-inactivation cycles.

Cell DIVE^TM^ was employed to generate HxIF image stacks of FFPE tissue microarrays from resected tissue samples from 432 chemo-naive CRC patients at single-cell resolution. This 55-dimensional spatial profiling of the patient-level tumor microenvironment served as input in our study. The 432 patient cohort was retrospectively acquired from Clearview Cancer Institute of Huntsville Alabama, and included Stage I through III CRC patients, who were followed between the years of 1993 and 2002. As shown in Supplementary Table 2, the median patient age and gender proportions were similar across all stages, with CRC recurring in 65 patients. The outcome distribution of the patients and their clinical attributes across the CRC stages are detailed in Supplementary Table 2. The use of chemo-naive (no administration of neoadjuvant or adjuvant therapies for the 5+ years of follow-up) CRC patient cohort provides SpAn the opportunity to interrogate unperturbed primary tumor biology.

### Recurrence-guided and spatially informed CRC prognosis

**Fig. 2:**
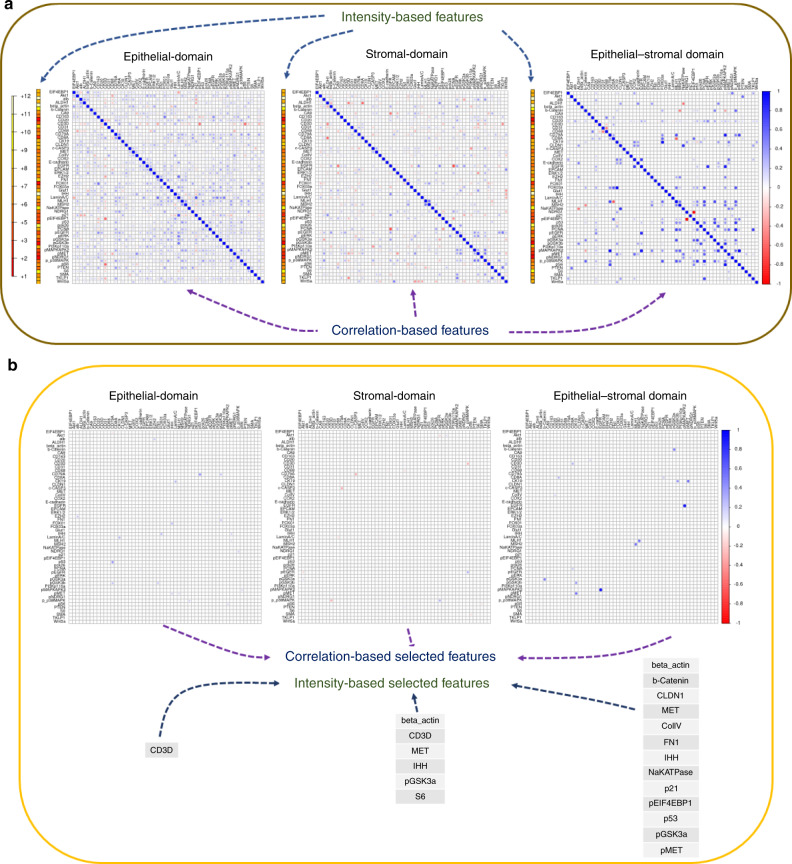
SpAn domain-specific feature selection. **a** Individual mean protein-expression intensity profile depicted as a vector and pairwise Kendall rank-correlations between protein expressions—visualized as a matrix for each of the three spatial domains. The protein expressions are shown in log scale. To prevent inclusion of false-positive protein expression, only intensities above the 85th percentile were considered as expressions and used to compute the correlations (see Methods). **b** Features, including both expressions and correlations, selected by SpAn based on L1-penalized Cox regression used for model selection. The selected features were consistently concordant at the 90% level with the recurrence outcome.

SpAn performs a virtual three-level spatial dissection of the tumor microenvironment, by first explicitly decomposing the TMA into epithelial and stromal regions as detailed in Methods and illustrated in Supplementary Fig. 3. The cells in the epithelial region are identified using E-cadherin cell–cell adhesion labeling and pan-cytokeratin, with individual epithelial cells segmented using a Na^+^K^+^ATPase cell-membrane marker, ribosomal protein S6 cytoplasmic marker, and DAPI-based nuclear staining. The resulting epithelial spatial domain of the TMA in Fig. 1a is shown in Fig. 1c. The remaining cells are assigned to the stromal domain and are visualized in Fig. 1d. These stromal cells have diverse morphologies^19^. Based on the epithelial and stromal domains, SpAn also identifies a third epithelial-stromal domain, shown in Fig. 1e, to explicitly capture a 100 µm boundary wherein the stroma and malignant epithelial cells interact in close proximity. Together these three intra-tumor spatial domains comprise the virtual three-level spatial dissection of the tumor microenvironment that forms the basis for the SpAn spatial model overviewed in Fig. 1f and detailed in Fig. 2.

**Fig. 3:**
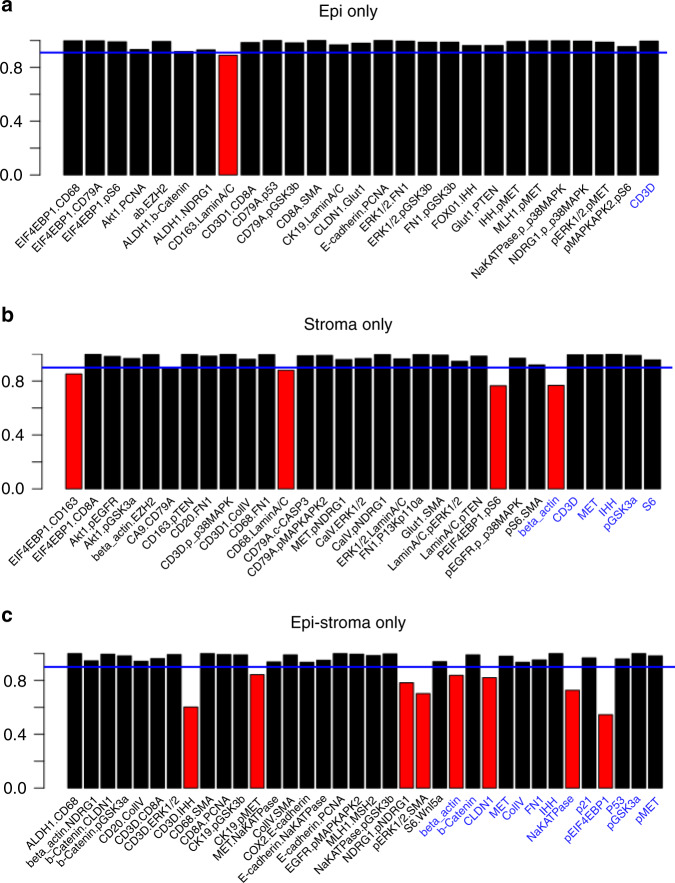
SpAn stability analysis. **a**–**c** Stability analysis of the selected features for each of the spatial domains, with only those features from the ones selected in Fig. 2b that maintain their sign in 90% of the 500 bootstrap runs are included as input into the SpAn spatial model. These features are visualized in black in the three bar graphs. Features selected in Fig. 2b that did not meet this criterion are shown in red. The blue solid line indicates the 90% threshold.

Utilizing expression of the 55 hyperplexed biomarkers, SpAn first computes the corresponding 55 mean intensities and 1485 Kendall rank-correlations as features characterizing each of the three spatial domains (see Fig. 2a). The mean intensity captures the average domain-specific expression profile of each biomarker, while the Kendall rank-correlations^22^ measure strength of association between any two biomarkers without presuming linearity (see Methods for details). Importantly, computation of domain-specific rank-correlations as explicit features for SpAn is used in place of the more typical approach of implicitly incorporating correlations as interactions between covariates (average biomarker expressions) within the prediction model^23^. These explicit features not only detect the association between two biomarkers presumably mediated by intracellular and intercellular networks all within the same spatial domain but also by mediators (e.g., exosomes) derived from another spatial domain. As an example, SpAn finds enrichment of KEGG ‘microRNAs in cancer’ pathway in the epithelial and epithelial-stromal domains (see below), while concurrently selecting correlation between CD163 and PTEN as a feature in the stromal domain for recurrence prognosis (see Fig. 3). As has been reported in gastrointestinal cancers, tumor cell-derived exosomal miRNAs mediate crosstalk between tumor cells and the stromal microenvironment, and induce polarization of the macrophages to the anti-inflammatory and tumor-supportive M2 state via activation of the PTEN-PI3K signaling cascade under hypoxic conditions resulting in enhanced metastatic capacity^24,25^.

SpAn then uses CRC-recurrence-guided learning to determine those specific spatial-domain features that constitute the optimal subset for prognosis via model selection based on L1-penalized Cox proportional hazard regression method (Fig. 2b)^26,27^. See Methods for details on penalized regression, Supplementary Fig. 4 for validity of the proportional hazard assumptions, and Supplementary Fig. 5 for determination of threshold for concordance with recurrence outcome. A follow-up analysis of the selected features is performed to test the stability of their contribution to recurrence prognosis through testing the stability of the sign of the corresponding coefficients at the 90% threshold. The final domain-specific features are shown in Fig. 3 and Supplementary Table 3 with additional details described in Methods.

**Fig. 4:**
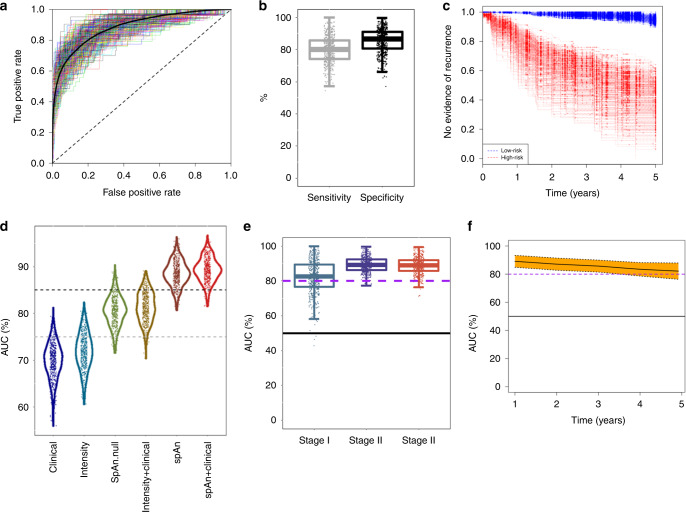
Performance of SpAn in predicting 5-year CRC-recurrence risk. **a** SpAn receiver operating characteristics (ROC) curves for predicting risk of 5-year CRC recurrence in patients with resected CRC primary tumor. The plot shows ROC curves, rendered in different colors for improved visual contrast, for 500 bootstrap runs with independent training and validation sets. Area under the mean ROC curve, shown as a black-solid curve, is 88.5% with a standard error of 0.1%. The black dashed 45-degree line indicates random guessing. **b** Boxplots of the sensitivity and specificity values for the same 500 bootstrap runs as in (**a**) with the operating point on the ROCs chosen by minimizing overall misdiagnosis rate. The mean sensitivity and specificity values, respectively, are 80.3% (standard error of 0.4%) and 85.1% (standard error of 0.3%). (Box plot center line: median value; box bounds: interquartile range (IQR); upper whisker: 3rd quartile + 1.5 IQR; lower whisker: 1st quartile − 1.5 IQR). **c** Kaplan–Meier recurrence-free survival curves for each of the 500 bootstrap runs for patients identified by SpAn at low and high risk of 5-year CRC recurrence. **d** AUC violin and boxplots of the bootstrapped ROC curves achieved by six CRC-recurrence prediction models. The models are (1) clinical model, (2) biomarker expression model (denoted by intensity), (3) SpAn.null model (denoting SpAn without spatial-domain context), (4) biomarker expression + clinical model, (5) SpAn, and (6) SpAn + clinical model. Five hundred bootstraps were used. Figure S8 lists the statistical significance of the pairwise performance comparisons between all the six models. The gray and black dashed lines represent ROC curves with AUC of 75% and 85%, respectively. **e** Boxplots of stage-based area under the 500 bootstrapped ROC curves showing the stable stage-based clinical performance of SpAn. The black-solid line indicates an AUC of 50% corresponding to random guessing. **f** Stable temporal performance of SpAn illustrated by the time-dependent AUC values plotted as a function of time in years. The 95% confidence interval computed using the 500 bootstraps is also shown by the yellow shaded area around the mean time-dependent AUC values depicted by the black-solid curve. The 0.8 and 0.5 AUC values are shown for reference by the purple-dashed and black-solid lines, respectively.

The coefficients that control the contribution of the selected features to each of the domain-specific models for assessing recurrence outcome were learned under L1 penalization and their values are, therefore, dependent on all 1540 features. To remove this dependence, SpAn relearns each of the three domain-specific model coefficients using L2 penalty in our penalized Cox regression model with only the optimally selected features as input. This L2-regularized learning allows SpAn to estimate optimal contribution of the selected features that are 90% concordant with the recurrence outcome. The resulting domain-specific coefficients are shown in Supplementary Fig. 6. As detailed in Methods, SpAn combines these domain-specific features weighted by their corresponding coefficients into a single recurrence-guided spatial-domain prognostic model, whose performance is shown in Fig. 4a. The results were obtained by bootstrapping (sampling with replacement) patient dataset to generate 500 pairs of independent training and testing sets using stratified sampling that ensured the proportion of patients in whom cancer recurred in each of the 5 years remained the same in each bootstrap. For each bootstrap, SpAn used the training data for learning and the independent testing data to compute the receiver operating characteristic (ROC) curve. These ROC curves are shown in Fig. 4a along with the mean ROC curve. The mean area under the curve (AUC) for bootstrapped ROC curves is 88.5% with a standard error of 0.1%, demonstrating the stable performance of SpAn. We also maximized Youden’s index^28^ to identify the clinically relevant operating point on the ROC curves that minimized the overall misdiagnosis rate. Figure 4b shows the resulting sensitivity and specificity values for all bootstrap runs, with mean values, respectively, of 80.3% (standard error of 0.4%) and 85.1% (standard error of 0.3%). High specificity limits SpAn from misidentifying no-evidence-of-disease patients as being at high risk of CRC recurrence, while at the same time good sensitivity allows SpAn to not miss high-risk patients. This is emphasized by a high positive likelihood ratio value of 7.2 (standard error of 0.23), which quantifies the large factor by which odds of CRC recurring in a patient go up, when SpAn identifies the patient as being at risk of CRC recurrence. At the same time a small negative likelihood value of 0.22 (standard error of 0.003) quantifies the decrease in odds of CRC recurrence in a patient when SpAn identifies the patient as being at low risk. Finally, these results are brought together in Fig. 4c, which show the large separation in recurrence-free survival curves of patients identified by SpAn at low and high risk of 5-year CRC recurrence.

### Validating the rationale behind SpAn

The rationale behind our “virtual-dissection followed by combination of the three specific spatial domains” approach is motivated by the acknowledged active role of the microenvironment and its spatial organization, and the differential role played by the epithelial and stromal domains in tumor growth and recurrence^2,13,29^. We tested the validity of this rationale within the context of our data by comparing the performance of SpAn with the null model, which is based on recurrence-guided learning of the spatially undissected patient TMA spot. We note that the learning procedure for the null model was identical to the domain-specific learning within SpAn. In addition, we also compared the performance of SpAn with four other models that included a clinical model, biomarker expression model, biomarker expression + clinical model, and SpAn + clinical model. The input into the clinical model were clinical features associated with age, gender and TNM stage, and the learning procedure was based on Cox proportional hazard regression^30^. The biomarker expression model input were biomarker expression intensities alone and the learning procedure was identical to SpAn. The biomarker expression + clinical, and SpAn + clinical models, respectively, combined biomarker expression and SpAn with the clinical model. Figure 4d shows the AUC violin and boxplots of the bootstrapped ROC curves achieved by each model. The figure illustrates the improvement SpAn achieves over the performance of other models. To quantify the statistical significance of this improvement, we performed Dunn’s pairwise multiple comparison post hoc analysis between the models based on non-parametric Kruskal–Wallis test^31^. Our analysis shows that the improvement in performance achieved by SpAn over all other models is statistically significant at the 99% confidence interval with a *p*-value much less than 0.005 (Supplementary Table 4). We specifically note that this is true for SpAn performance in comparison to the null model based on the spatially undissected patient TMA spot without spatial-domain context. This improved performance of SpAn highlights the importance of explicitly modeling the epithelial, stromal and epithelial-stromal spatial domains associated with the TME. Interestingly, beyond supporting our rationale, this comparative test also demonstrates that joint utilization of biomarker expressions and their correlations results in superior performance of both SpAn and its null model over clinical features and biomarker expressions alone (also see Supplementary Fig. 7). We note that published state-of-the-art approaches that include Immunoscore®^14,15^ rely on biomarker expressions. Finally, we observe that the marginal performance improvement over SpAn achieved by including clinical features with SpAn—the SpAn + clinical model—is not statistically significant with a *p*-value of 0.082.

### SpAn predicts 5-year recurrence in Stages I–III CRC patients

The ability to identify patients in whom CRC will recur, especially for those patients in Stages II and III of tumor progression is highly clinically relevant. Figure 4e shows that, although the modeling of SpAn is TNM stage-independent, SpAn can consistently identify patients in whom risk of CRC recurrence is high for Stages I through III, with mean AUC of bootstrapped ROC curves for the three stages, respectively, being 82.1%, 89.4%, and 88.6%. Standard error of these mean AUC values, respectively, is 0.4%, 0.2%, and 0.2%, demonstrating the stability of SpAn performance. Although the overall performance across all three stages is highly significant with the potential of improving prognosis, the relative reduction in Stage I performance may be a consequence of the small cohort of only ten patients in Stage I with CRC recurrence.

The ability of SpAn to predict risk of recurrence in individual patients from all three Stages, is relevant in the context of administering adjuvant therapy, especially for Stage II patients. Current guidelines for treating Stage II CRC patients from The National Comprehensive Cancer Network (NCCN)^32^, the American Society of Clinical Oncology (ASCO)^33^, and the European Society of Medical Oncology (ESMO)^34^ do not recommend routine adjuvant chemotherapy for Stage II patients, but do state that it should be considered for sub-population of Stage II patients that are at higher risk and might benefit from being put on adjuvant therapy regimen^35^. The personalized prognostic potential of SpAn implies that we could triage Stage II patient cohorts into low and high-risk groups, with the latter being further considered for therapy. Furthermore, SpAn could help with postoperative surveillance of high-risk Stage II patients with more intensive follow-up regimes^36^.

While 20–30% of Stage II CRC patients are at high risk of recurrence, there are Stage III patients that have good prognoses of stable 5-year recurrence-free survival. SpAn, therefore, could also be used to fine-tune their postoperative surveillance and adjuvant chemotherapy regimens.

### Prognostic performance of SpAn remains stable over 5 years

A majority of CRC recurrence occurs in the first 5 years, with 90% occurring in the first four^37,38^. We, therefore, consider the time-dependent performance^39^ of SpAn during the first 5-year period. Figure 4f plots the AUC for time-dependent ROC performance. The performance of SpAn in predicting risk of recurrence remains consistent and stable (95% confidence interval shown) with only a small, and gradual reduction in time-dependent AUC values as we move away from the resection and imaging timepoint. This result suggests SpAn captures the critical biological underpinnings of recurrence in the primary tumor. Supplementary Fig. 8 shows the time-dependent AUCs for domain-specific temporal performance of SpAn.

### SpAn infers spatial-domain networks underlying CRC recurrence

**Fig. 5:**
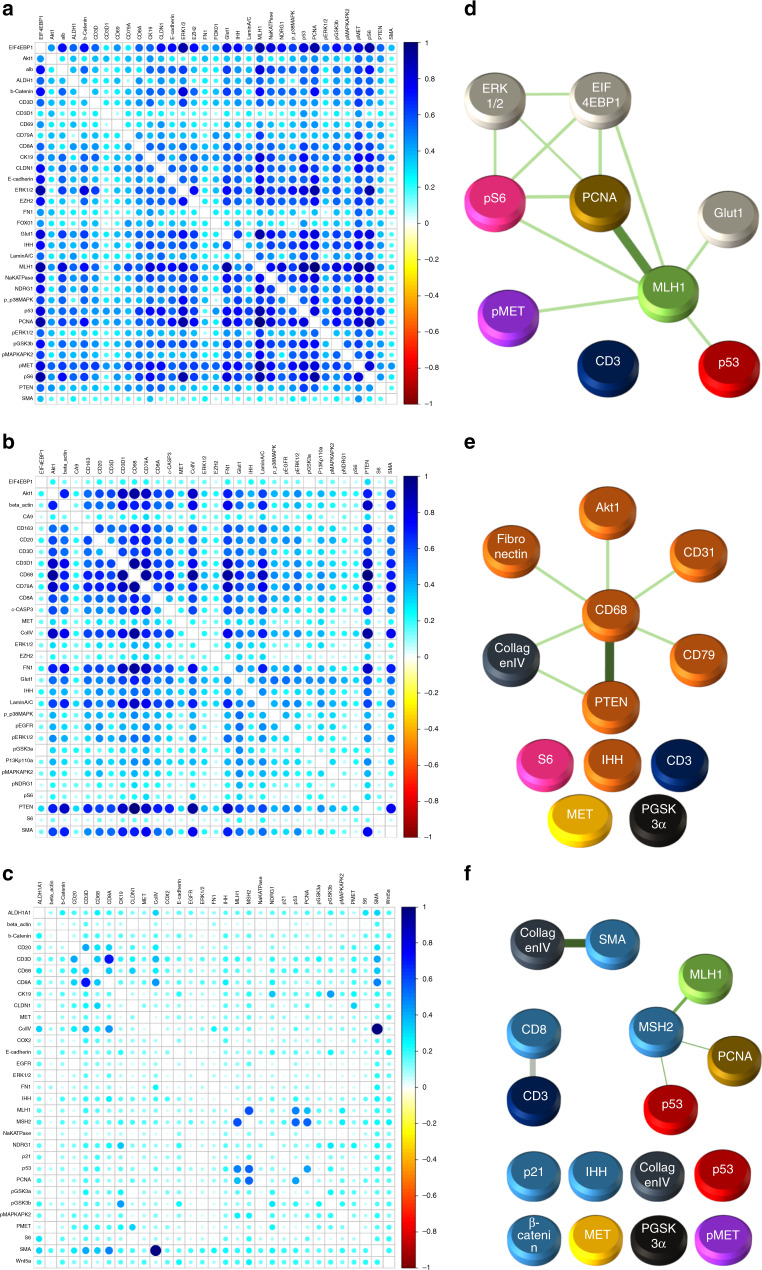
SpAn derived spatial-domain networks. **a** Epithelial domain, **b** Stromal domain, and **c** Epithelial-stromal domain Jensen–Shannon divergence matrices that show the information distance of partial correlations (computed for biomarkers selected by recurrence-guided SpAn feature selection and stability analysis) between patients in the no-evidence-of-CRC and CRC-recurrence cohorts. **d** Epithelial domain, **e** Stromal domain, and **f** Epithelial-stromal domain spatial-domain networks obtained by thresholding the corresponding spatial-domain information distance matrices at the 99th percentile, which identify differential change most significant for CRC-recurrence prognosis. (The graphical and color rendering is to ensure ease of visualization.) All three spatial-domain networks include disconnected subnetworks with isolated nodes. These nodes correspond to biomarkers selected by SpAn as indicated in blue in Fig. 3. Biomarker expression is an intrinsic property that, unlike correlations, does not describe relationships between different biomarkers, and therefore, is naturally expressed by an isolated node without any connecting edge.

Given the high prognostic performance of SpAn, we took a systems perspective to understand and to explain the underlying network biology responsible for this performance within each of the three spatial domains. For each domain, we quantified the unique associations between biomarkers included in the selected features through partial correlations between every biomarker pair, when controlling for other biomarkers as described in Methods. This approach was performed for all patients. The resulting partial correlation for every biomarker pair was separated into two groups according to no-evidence-of-CRC and CRC-recurrence patient cohorts and the information distance based on Jensen–Shannon divergence^40^ was computed between them (see Methods for more details). The resulting domain-specific distance matrices, shown in Fig. 5a–c, define associated graphs with the nodes being the biomarkers and edge weights quantifying the differential change, the information distance, in biomarker association between patients in which CRC recurred and those in which there was no evidence of recurrence. The stronger the weights, the larger the distance, and the more significant the differential change in association between the two markers for the two patient cohorts. We defined the graphs generated by the distance matrices thresholded at the 99th percentile as the spatial-domain networks that were most significant for CRC-recurrence prognosis. Figure 5d–f shows the resulting networks for the three spatial domains that reveal the heterogeneous nature of the cell populations and signaling pathways leveraged by SpAn in CRC-recurrence prognosis.

The epithelial-stromal domain network is comprised of three dominant subnetworks associated with tumor-invading T lymphocytes^41^, disruption in DNA mismatch repair cellular process, and the role of cancer-associated fibroblasts (CAFs) in the desmoplastic microenvironment as indicated by the strong edge weight between smooth muscle actin (SMA) and collagen IV. CAFs are well-known to promote EMT^42^ and the differential expression of beta-catenin and phosphorylated-MET in Fig. 5f is also consistent with the epithelial-stromal domain supporting the mesenchymal phenotype^43^. These features have also been identified with those distinguishing consensus molecular subtype (CMS) 4 that is associated with a poor prognosis in comparison to the other 3 subtypes in the transcriptome-based classification^11,12^. Interestingly, the epithelial-stromal spatial domain also reveals the presence of DNA mismatch repair network that has been associated with regulation of T-lymphocyte infiltration, a prominent feature of CMS1. Thus, the epithelial-stromal spatial domain associated with recurrence combines two features, where in contrast, each alone is associated with two different CMS subtypes. This theme extends to the epithelial spatial domain in Fig. 5d, where metabolic deregulation, a prominent feature of CMS3, and DNA mismatched repair, a hallmark of CMS1 are evident. The association of these two subnetworks in the epithelial domain has the potential to promote tumor cell growth while escaping immune surveillance. Finally, we observe a prominent tumor associated macrophage (TAM) network in the stromal spatial domain (Fig. 5e). TAM polarization toward the M2 phenotype regulated by AKT/PTEN has been associated with poor prognosis in CRC that could result from their immunosuppressive and matrix remodeling phenotypes^25^.

### Spatial-domain networks reveal domain-specific CRC network biology

**Fig. 6:**
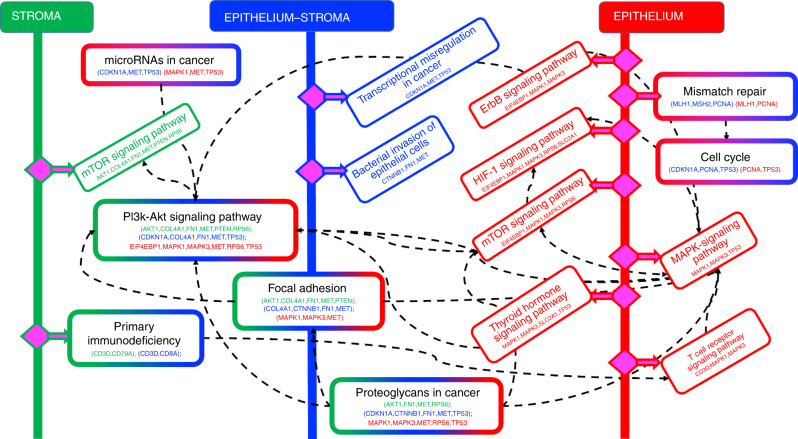
CRC-recurrence-specific network biology inferred by SpAn. Domain-specific biomarkers identified by the spatial-domain networks are used to interrogate the KEGG and STRING databases to identify domain-specific pathways enriched by the biomarkers. The epithelial, stromal, and epithelial-stromal domain are, respectively, shown in green, red, and blue, with pathways unique to those domains also coded with the same colors. Pathways that are enriched in more than one domain are coded with a color combination of those respective domains. For example, the PI3K-AKT signaling pathway is enriched in all three spatial domains, and therefore, has a boundary box color-coded with all three colors. On the other hand, the mismatch repair pathway is enriched in the epithelial and epithelial-stromal domains, and is therefore, color-coded by red and blue colors.

We used STRING^44^ and KEGG^45^ databases to identify pathways enriched by biomarkers within each of the spatial-domain networks and further corroborate their connections to prominent features in the CMS subtype classification. Figure 6 shows the pathways enriched in each of the three spatial domains, and further identifies those that are common to a majority of at least two of the three spatial domains. Since their identification is based on the spatial-domain networks we computationally identified as significant for CRC-recurrence prognosis, these pathways play a differentially important role in prognosis of CRC recurrence.

CMS2 tumors are associated with chromosomal instability pathway and enrichment of genes associated with cell cycle and proliferation. Interestingly, both Pi3k-Akt signaling and cell cycle pathways enriched in our analysis are associated with CMS2 tumor subtype, with almost 60–70% of CRCs associated with dysregulation of Pi3k-Akt signaling pathways^46^.

Tumors associated with the CMS4 mesenchymal phenotype show upregulated expression of genes involved in epithelial-to-mesenchymal transition along with increased stromal invasion, angiogenesis and transforming growth factor-β (TGF-β) activation^11,12^. Interestingly, proteoglycans in cancer, focal adhesion and microRNAs in cancer pathways enriched in our analysis enable the mesenchymal phenotype. For example, non-coding microRNAs both regulate and are targets of upstream regulators for modulating the epithelial-to-mesenchymal phenotype by targeting EMT-transcription factors such as ZEB1, ZEB2, or SNAIL^47^. Similarly, the focal adhesion pathway through the integrin family of transmembrane receptors mediates attachment to the extracellular matrix, and when dysregulated promotes cell motility and the mesenchymal phenotype^48,49^. Furthermore, extracellular and cell surface proteoglycans with their interaction with cell surface proteins such as CD44 have been known to promote tumor cell growth and migration^50,51^.

Our analysis suggests that by capturing correlation-based crosstalk between heterocellular signaling pathways, SpAn leverages the interconnections between the subtypes for a high performing CRC-recurrence prognosis and reveals a synergistic role of the CMS subtypes in CRC progression and recurrence. We note that the ability of SpAn to leverage these interconnections is due to the spatial-context-preserving sampling of a diverse set of CRC-relevant biomarkers enabled by HxIF imaging.

Interestingly, this network biology paradigm also shows enrichment of pathways specific to a single spatial domain whose oncogenic or tumor suppressive roles in CRC is an active area of research but whose differential role in CRC recurrence has not been widely studied. For example, in the epithelial domain our analysis shows the enrichment of Thyroid hormone signaling pathway that has been associated with a tumor suppressive role in CRC development^52,53^. In contrast, the bacterial invasion pathway, enriched in epithelial-stromal boundary region, has been implicated in the oncogenic role of the colonic microbiome in CRC development^54,55^.

Our analysis also reveals enrichment of certain other pathways, such as the hypoxia-inducible factor 1 (HIF-1), human epidermal growth factor receptor 2 (HER2) and T-cell receptor signaling pathways in the epithelial domain. Hypoxia is typical in many solid tumors in CRC with HIF-1 regulating tumor adaptation to hypoxic stress^56^. Alterations in Her-2 signaling, either through genomic amplification or mutations is tumor promoting, and anti-HER2 therapies for preventing CRC recurrence and are a focus of on-going work^57^. We finally note that MAPK and PI3K-AKT signaling cascades are implicated in many of the above discussed pathways.

## Discussion

**Fig. 7:**
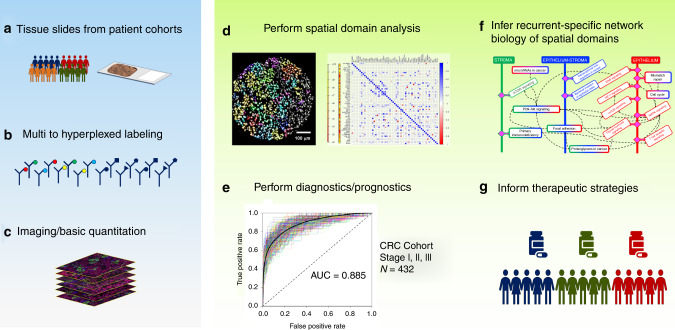
Workflow of SpAn computational and systems pathology platform. **a–c** SpAn utilizes images of resected primary tumors from TMAs or whole slide images based on hyperplexed fluorescence and other imaging modality platforms^18–21^ to **d** perform spatial-domain analysis for **e** patient diagnoses and prognoses and **f** infer recurrence-specific spatial-domain networks to **g** potentially inform therapeutic strategies.

This study highlights the importance of spatial context of the primary tumor microenvironment in conferring distinct malignant phenotypes such as recurrence in CRC. We show how a computationally unbiased approach can be implemented through statistical modeling of spatially defined domains leading to a highly specific and sensitive platform for prognostic and diagnostic tests, as well as potentially inferring therapeutic strategies (Fig. 7). Although type, density and location of immune cells within CRC tumor samples have previously been utilized to predict patient outcome^58^, SpAn is novel in concept and distinct in approach for a few inter-related reasons. Unlike studies that are association-based, for example, associating recurrence with immune profiling of the CRC tumor^58^, SpAn is an outcome-driven method that utilizes the recurrence outcome to implement a systems approach to both predict CRC recurrence in patients and infer domain-dependent network biology most significant for this prediction. This outcome-driven systems approach, therefore, allows formal modeling of CRC recurrence as an emergent phenotype of the underlying TME, its spatial context and its molecular and cellular diversity. Furthermore, identifying spatial-domain networks that capture differential change most significant for recurrence, allows SpAn to be a hypothesis generating systems pathology platform that provides testable hypotheses regarding how spatial association of common networks could potentially lead to emergent signaling networks that confer malignant phenotypes in CRC patients. For example, an epithelial domain network coupling cell metabolism and DNA repair is consistent with tumor cell growth at the expense of T-cell exclusion and functional deficiency^11,12^ (Figs. 5 and 6). Likewise, the hijacking of CAFs to support EMT in the context of diminished immune surveillance in the epithelial-stromal spatial domain^42,59,60^ (Figs. 5 and 6) and the PI3K/AKT-mediated polarization of TAMs within the stromal domain (Figs. 5 and 6) can conspire to facilitate migration of tumor initiating cells to promote both local and distant recurrence^61^.

SpAn, when used in combination with a non-destructive hyperplexed imaging platform such as Cell DIVE^TM^ allows mechanistic hypotheses to be tested through iterative probing of the same spatial domains with additional biomarkers inferred by the pathway analyses (Figs. 6 and 7). We expect even more specific mechanistic biomarkers to emerge based on the iterative hyperplexed imaging approach that incorporates finer stage-based focus, thereby reducing the total number of biomarkers needed for optimal analyses. We will be pursuing this in subsequent studies. This feature of SpAn combined with a hyperplexing imaging platform will not only allow refinement of its prognostic ability, but since the iterative analysis can be potentially conducted in real time with further advances in the technologies, it may allow the prognosis to be specific to individual patients. Importantly, by enabling the testing of mechanistic hypotheses in patient samples directly connected to a specific clinical outcome, SpAn may inform therapeutic strategies to prevent the outcome. For example, immunotherapy has shown benefits in microsatellite instable (MSI) CRC patients but remains refractory in microsatellite stable (MSS) CRC patients^62^. By combining non-destructive hyperplexed imaging with iterative analysis, SpAn has the potential to identify spatial-domain networks that are differentially expressed  in MSI and MSS CRC patient cohorts. The next phase of this work will take advantage of sampling multiple regions of primary tumors with larger TMAs and/or whole side sections, exploring other spatial analytics ranging from simple to sophisticated spatial heterogeneity metrics^63^ and incorporating a combination of protein and nuclei acid biomarkers^64^.

The ability of SpAn to exploit spatial context of the tumor makes it suitable to study cancers that progress via spatially mediated signaling interactions with their TME. SpAn, therefore, is applicable to solid tumors including sarcomas, carcinomas, and lymphomas, which co-evolve with the TME^65^ of their abnormal tissue mass. We will pursue applications of SpAn to solid tumors beyond colorectal adenocarcinoma in subsequent studies. Our present retrospective study provides the foundation for such studies in other solid tumors. It also establishes feasibility of implementing SpAn in prospective studies predicting disease outcomes in patients with CRC and other malignant solid tumors. The high specificity and sensitivity of SpAn lies in its ability to unbiasedly identify emergent networks that appear to be closely associated and likely to be mechanistically linked to recurrence. We anticipate that hyperplexed datasets based on multiple imaging modalities will be generated faster and become less expensive as the technology evolves to become a mainstay tool to analyze solid tumors.

## Methods

### Patient cohort and tissue microarray (TMA)

As detailed in Gerdes et al.^19^, the CRC cohort in this analysis was collected from the Clearview Cancer Institute of Huntsville Alabama from 1993 until 2002 with 747 patient tumor samples collected as paraffin-embedded specimens. Tissue microarrays were constructed by Applied Genomics (later part of Clarient Lab) to facilitate large scale biomarker analysis. Cores with 0.6-mm diameters from the patient samples were distributed across seven slides. After quality control measures were taken, 694 TMA patient spots remained for analysis. Sample attrition was due to insufficient tumor fraction in the representative TMA core. Of the remaining samples, 450 were chemo-naive CRC patients that were treated with surgery alone, and the remaining 244 patients were treated with 5-fluorouracil-based chemotherapy regimens. Four-hundred and thirty-two chemo-naive patients were used in this study. Supplementary Table 2 details the median age, gender, recurrence, recurrence time, and survival times for the 432 Stage I–III CRC patients. As can be seen the patient cohort is balanced in age and gender across the three stages.

### Antibody validation

We ensured that the correct biomarker expression was captured by the imaging system using an antibody standardization process^19^. Specifically, antibodies were selected based on their staining specificity and sensitivity, compatibility with the two-step antigen retrieval, and resilience during 1, 5, and 10 rounds of dye-inactivation chemistry. Depending on the marker, a variety of specificity tests were conducted including, immunogen peptide blocking before incubation with tissue, drug-treated fixed cell lines, fixed cell lines with gene amplification or deletion, phosphatase treatment of samples to verify phospho-specificity, and visual inspection by expert pathologists of expected localization patterns. Furthermore, fluorescent dyes were conjugated to the primary antibody at several initial dye substitution ratios and specificity of each conjugate was verified and sensitivity compared with levels found in previous experiments. Staining performance was assessed by expert biologists and poor or non-specific staining was excluded.

### Cell DIVE-based hyperplexed imaging (HxIF) of tissue microarrays (TMA)

The 55 biomarkers plus DAPI nuclear counterstain included in this study are described in Supplementary Fig. 2 and Supplementary Table 1. HxIF imaging of a TMA slide was performed using sequentially multiplexed labeling and imaging of 2–3 biomarkers along with DAPI counterstain through a label–image–chemical-inactivation iterative cycle^19^ visualized in Supplementary Fig. 1 and detailed in Supplementary Table 5. Broadly, the supporting information details the hyperplexed immunofluorescence workflow with information on iterative cycles of antibody labeling of single 5 µm formalin-fixed and paraffin-embedded tissue sections and TMA slides, autofluorescence removal, imaging, and dye inactivation in tissue. All samples were stained and imaged in a single batch for 2–3 biomarkers and DAPI at a time.

### Image processing and single-cell analysis

DAPI-based nuclear staining was used to register and align sequentially labeled and imaged TMA spots prior to downstream image analysis steps^19^. Autofluorescence was removed from the stained images^19,66^, which were then segmented into epithelial and stromal regions (Supplementary Fig. 3), differentiated by epithelial E-cadherin staining. This was followed by segmentation of individual cells in both the epithelium and stroma. Epithelial cells were segmented using Na^+^K^+^ATPase-based cell-membrane staining to delineate cell borders and membrane regions, the cytoplasmic ribosomal protein S6 for cytoplasm identification, and DAPI stain for nuclear regions. Protein-expression level and standard deviation were subsequently quantified in each cell. The epithelial-stromal domain was identified via a three-step process. First, a tessellation of the patient TMA spot was performed using partially overlapping circles with a diameter of 50 µm. Second, only those circles with both positive and negative E-cadherin staining were retained. Finally, union of these circles resulted in a contiguous epithelial-stromal domain with a width of 100 µm.

### Quality checks and data normalization

Following single-cell segmentation, several data pre-processing steps were conducted. These included cell filtering, spot exclusion, log2 transformation and slide to slide normalization. Cells were included for downstream analysis if their size was greater than 10 pixels at ×20 magnification. The hyperplexing process can result in the tissue being damaged, folded, or lost. Image registration issues can also result in poor-quality cell data. Therefore, a tissue quality index based on the correlation of that image with DAPI was calculated for each cell for each round. Only those cells whose quality index equals to or greater than 0.9 (meaning that at least 90% of the cells overlapped with DAPI) were included. All the slides for all the biomarkers were adjusted to a common exposure time per channel. The data were then log2 transformed. A median normalization that equalizes the medians of all the slides was performed to remove slide to slide non-biological variability.

### SpAn input features

For each of the epithelial, stromal, and epithelial-stromal spatial domains, SpAn used *M* = 1540 domain-specific biomarker feature vector **f** as input. This input feature vector comprised of (1) mean intensity value of 55 biomarkers averaged across all cells within the spatial domain, and (2) 1485 (=55*54/2) Kendall rank-correlations between all 55 biomarker pairs. Kendall rank-correlation was chosen as the correlation metric because it is a non-parametric measure of association between two biomarkers. Moreover, its use of concordant and discordant pairs of rank-ordered biomarker expression for computing correlation coefficients allows it to robustly capture biomarker associations in presence of measurement noise and small sample size. Rank-correlation for each pair of biomarkers was computed for each spatial domain from all cells across the spatial domain expressing the biomarkers. This approach is distinctly different from prediction models that typically consider correlations via interactions, implicit within the models, between mean biomarker intensity expressions—with the biomarker expressions being the only covariates of the model^23^. We emphasize that we did not compute correlations through mean intensity biomarker expression across the spatial domain, but instead used biomarker expressions across individual cells of the spatial domain to explicitly compute domain-specific rank-correlation values between every pair of biomarkers to form the SpAn correlation feature set.

Before computing these two sets of features, SpAn analysis workflow included an initial intensity threshold step to ensure feature robustness. Specifically, we computed intensity-based distribution of cell-level biomarker expression separately for every biomarker across each patient TMA spot. Only intensities above the 85th percentile on this distribution were considered as biomarker expression and included in computing the intensity features. This focus on the right-tail of the intensity distribution was deliberately conservative, and although it might have potentially excluded low-intensity biomarker expression, it minimized inclusion of false-positive expressions into the analysis.

### Penalized Cox proportional hazard regression

For each spatial domain, SpAn implemented the Cox proportional hazard model via the partial likelihood function $L\left( {\mathbf{\beta }} \right) = \mathop {\prod}\nolimits_{k = 1}^K {\frac{{e^{\left( {{\mathbf{f}}_{i_k}^{\mathrm{T}}{\mathbf{\beta }}} \right)}}}{{\mathop {\sum }\nolimits_{i \in R_k} e^{\left( {{\mathbf{f}}_i^{\mathrm{T}}{\mathbf{\beta }}} \right)}}}}$ with the penalty given by $P_{\lambda ,\alpha }\left( {\mathbf{\beta }} \right) = \mathop {\sum}\nolimits_{m = 1}^M \lambda \left( {\alpha \left| {\beta _m} \right| + \frac{1}{2}\left( {1 - \alpha } \right)\beta _m^2} \right)$, and *α* = {0,1}. (The validity of using the Cox proportional hazard regression model is demonstrated in Fig. S4.) Given feature vector **f** as input, the partial likelihood *L*(**β**) quantifies the conditional probability of observing CRC recur in a patient at time *t*_*k*_ (proportional to the numerator $e^{\left( {{\mathbf{f}}_{i_k}^{\mathrm{T}}{\mathbf{\beta }}} \right)}$ of *L*(**β**)), given the risk that a patient will recur from the set *R*_*k*_ of patients at risk at time *t*_*k*_ (proportional to the denominator $\mathop {\sum}\nolimits_{i \in R_k} {e^{({\mathbf{f}}_i^{\mathrm{T}}\beta )}}$), over all time *t*_*k*_, *k* = 1,…,*K*, as quantified by the product over time index *k*. The partial likelihood is a function of the coefficient vector **β**, whose penalized estimate is then used to compute the proportional hazard ratio HR = $e^{\left( {{\mathbf{f}}^{\mathrm{T}}\beta } \right)}$. SpAn computed this estimate via a two-step process that first selects the parsimonious set of features required for optimally predicting the risk of recurrence, and then learns the model predicting the risk of recurrence based on the selected features.

The feature selection step is implemented via L1-penalized (LASSO) Cox regression where *α* is set to 1 in penalty *P*_*λ*,*α*_(**β**). LASSO-based L1-penalized model selection performs feature selection by forcing the coefficients of vector **β** that play a minimal role in predicting risk of recurrence to zero. This is done in a principled manner by minimizing the model deviance along the LASSO regularization path^27,67^. The features corresponding to the non-zero coefficients in **β** are the features selected by SpAn to define the final functional form of Cox proportional hazard model. Model learning based on this functional form is performed in the second step via maximizing the partial likelihood function with L2-regularization as the penalty, implemented by setting *α* to 0 in the penalty term^27,67^. L2-regularization allows SpAn to learn the Cox proportional hazard model while avoiding over-fitting. An advantage of this two-step process is the decoupling of feature selection from estimation of beta coefficient values, resulting in the latter not being conditioned on the complete set of 1540 features but being dependent only on the selected features.

To ensure the stability of the selected features, SpAn repeated model selection over 500 bootstraps, and included only those features that were consistently concordant at the 90% level with the recurrence outcome. (The rationale for 90% concordance is discussed in Supplementary Fig. 5.) SpAn next performed a stability check on the beta-coefficients estimated in the second step. Specifically, the stability of the coefficient sign in 90% of the 500 bootstrap runs was tested, and only features that passed this threshold (Fig. 3) were included in the spatial-domain model. SpAn performed this process independently for each of the three spatial domains resulting in domain-specific recurrence-guided features (Fig. 3) and their coefficients (Fig. S6).

### SpAn is computationally unbiased

SpAn begins penalized Cox proportional hazard regression by including the full 1540 features. It then utilizes LASSO-based shrinkage to parsimoniously optimize the full model along the L1 regularization path by minimizing model deviance^67^. By combining this principled shrinkage via L1-penalized Cox proportional hazard regression, with bootstrapping to establish the stability of the selected subset of features at 90% concordance with the recurrence outcome (Supplementary Fig. 5), SpAn avoids typical biases associated with many model selection approaches based on stepwise variable selection, backward elimination, and forward selection^68^. These biases include *R*^2^ values being biased high, F and *χ*^2^ test-statistics not having their associated distributions, *p*-values being biased toward zero, and standard errors of regression coefficient estimates being biased low, while absolute values of regression coefficients being biased high.

### SpAn is robust to dataset imbalance

Many machine learning algorithms underpinning prediction models can potentially result in suboptimal performance for imbalanced datasets, where the number of resected CRC patients with no evidence of disease in the first 5 years (low risk) is not similar in number to patients in whom CRC recurred within the first 5 years (high risk). SpAn, however, is relatively robust to this imbalance due to its use of Cox proportional hazard model for predicting 5-year CRC recurrence. Cox proportional hazard model is based on the hazard function which utilizes the conditional probability of CRC recurrence in a patient at time *t*, given a risk set at that time and the knowledge that CRC has not recurred in the patient until time *t*^27^. Therefore, in Cox proportional hazard model the timing is more critical than the number of recurrences per se. As an example, consider the hypothetical scenario, where, instead of actual 65 high-risk CRC patients in the study, all 432 CRC patients in the cohort were high risk, with no patient at low risk. The Cox proportional hazard model will, in principle, remain valid because it models the conditional rate of CRC recurrence as a function of time and not the number of recurrences themselves. However, the SpAn workflow does ensure that in this study the size of the high-risk CRC patients is large enough for us to be able to sample high-risk CRC patients in each recurrence risk set corresponding to each of the 5 years. Supplementary Table 6 illustrates a typical stratified sampling result employed by SpAn to construct the training and testing sets. As can be seen this stratified sampling not only ensures that high-risk patients are approximately equally distributed between the training and testing datasets, but it also ensures that high-risk CRC patients are captured in each recurrence risk set corresponding to each of the 5 years. Thus, SpAn implements a form of risk set sampling^69^. It is also instructive to note that, as highlighted by CRC epidemiological studies^37,38^, our CRC patient cohort along with our sampling strategy captures the real-world trend that a majority of CRC recurrence in patients occurs in the first 5 years of primary tumor resection.

### Spatial model

Each of the three recurrence-guided domain-specific models defined a hazard risk given by $e^{\left( {{\mathbf{f}}_{{\mathrm{epithelial}}}^{\mathrm{T}}{\mathbf{\beta }}_{{\mathrm{epithelial}}}} \right)}$, $e^{\left( {{\mathbf{f}}_{{\mathrm{stromal}}}^{\mathrm{T}}{\mathbf{\beta }}_{{\mathrm{stromal}}}} \right)}$, and $e^{\left( {{\mathbf{f}}_{{\mathrm{epi}} - {\mathrm{stromal}}}^{\mathrm{T}}{\mathbf{\beta }}_{{\mathrm{epi}} - {\mathrm{stromal}}}} \right)}$ for the epithelial, stromal, and epithelial-stromal domains, respectively. SpAn then defined the final overall risk of recurrence model as $\prod_{s \in S} e^{\left( {{\mathbf{f}}_s^{\mathrm{T}}\beta _s} \right)}$, with *S* = {epithelial, stromal, epi–stromal}.

### Partial correlations and spatial-domain networks

For each spatial domain, the selected features identified a set of biomarkers specific to predicting risk of CRC recurrence. SpAn used them to define a space of biomarkers within which partial correlations between every pair was computed by controlling for confounding effect of biomarkers not defining the pair^70^. The process performed on each patient was as follows: Let the set of biomarkers identified by the selected features be *N* (≤55). Using the already computed Kendall rank-correlations between the 55 biomarkers, an *N* × *N* correlation matrix **C** corresponding to the *N* biomarkers was constructed, with small shrinkage-based modification to guarantee its positive definiteness, and therefore, its invertibility. Next, the *N* × *N* precision matrix **P** was computed by inverting **C**. The partial correlation between any two biomarkers bm_*i*_ and bm_*j*_ within the set identified by the selected features, was then computed using $\rho _{{\mathrm{bm}}_i,{\mathrm{bm}}_{\mathrm{j}}} = \frac{{ - p_{{\mathrm{bm}}_i,{\mathrm{bm}}_j}}}{{\sqrt {p_{{\mathrm{bm}}_i,{\mathrm{bm}}_i} \cdot p_{{\mathrm{bm}}_j,{\mathrm{bm}}_j}} }}$, where $p_{{\mathrm{bm}}_i,{\mathrm{bm}}_j}$ is the (*i*,*j*)th element of the precision matrix **P**. The partial correlations were performed for all patients and were then separated into two groups corresponding to patients with no evidence of disease and those patients in which CRC recurred. Probability distributions of the partial correlations—on the compact set [−1,1]—within each group were computed and the information distance between these two distributions was computed using the Jensen–Shannon divergence. This information distance defines the differential change in the association—partial correlation—between biomarkers bm_*i*_ and bm_*j*_ in the two patient cohorts. Greater the distance, larger the differential change. Repeating this process for all *N*(*N*−1)/2 biomarker pairs resulted in the information distance matrices shown in Fig. 5a–c for the three spatial domains. These information distance matrices were thresholded at the 99th percentile resulting in the computationally inferred spatial-domain networks shown in Fig. 5d–f. The high percentile was chosen to ensure that most discriminative networks are captured.

### Enrichment analysis

The STRING database^44^ was queried with the set of proteins identified by the spatial-domain networks generated by thresholding the domain-specific information distance matrices at the 99th percentile, to perform functional enrichment analysis using Fisher’s exact test with multiple testing correction.

### Reporting summary

Further information on research design is available in the Nature Research Reporting Summary linked to this article.

## Supplementary information

Supplementary Information

Reporting Summary

## Data Availability

Due to the multi-terabyte size of the dataset, the data that supports the findings of this study can only be made available upon reasonable request.

## Code availability

 The computational and systems pathology intellectual property is owned by the University of Pittsburgh and is exclusively licensed to SpIntellx Inc., Pittsburgh, PA as TumorMapr™. Use of TumorMapr code requires a licensing agreement with SpIntellx (info@spintellx.com).
