# Supplementary material for: Spatial domain analysis predicts risk of colorectal cancer recurrence and infers associated tumor microenvironment networks
Source: Nat Commun. 2020 Jul 14;11:3515. doi: 10.1038/s41467-020-17083-x (PMC7360741; doi:10.1038/s41467-020-17083-x)
Supplement: Supplementary file 1 — Supplementary Information [file 41467_2020_17083_MOESM1_ESM.pdf]

Supplementary Information for

Spatial domain analysis predicts risk of colorectal cancer recurrence and infers associated tumor microenvironment networks

Uttam et al.

This PDF file includes:

1. Supplementary Figures 1 - 8
2. Supplementary Tables 1- 6

Supplementary Figures

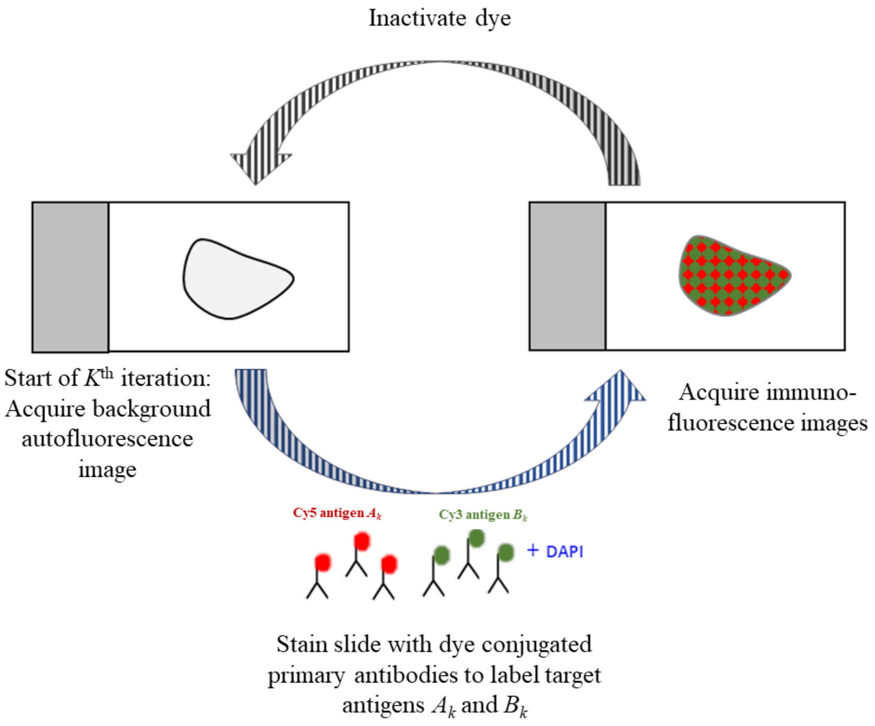

Supplementary Figure 1. Cell DIVE HxIF imaging and processing scheme. For the Kth iteration, autofluorescence image of the tissue is acquired prior to labeling it with 2-3 fluorescent dye-conjugated primary antibodies and DAPI counterstain. Fluorescent-labeled tissue images are then acquired, followed by inactivation of the dyes and start of the next iteration. (See Supplementary Table 5 for details on the iterative imaging cycles.)

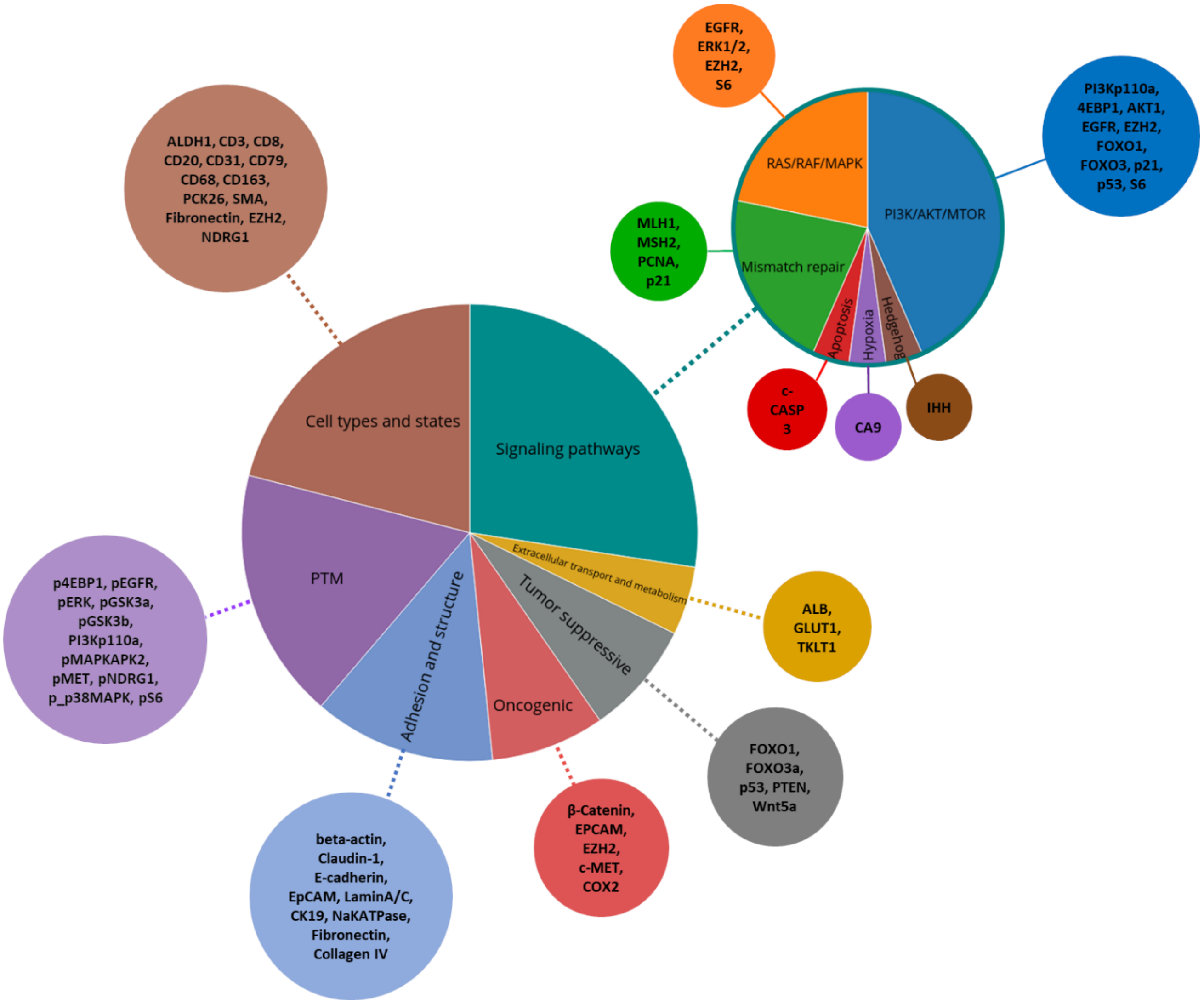

Supplementary Figure 2: Biomarker selection and organization. Fifty-five biomarkers along with DAPI were imaged using the Cell DIVE platform. The biomarkers were selected from seven broad categories that include **1**. biomarkers sampling the network biology of signaling pathways (PI3K/AKT/MTOR[1], RAS/RAF/MEK[2], Mismatch Repair[3] (MMR), Hedgehog signaling[4] , Hypoxia-signaling[5], Apoptosis[6]); **2**. biomarkers associated with extracellular transport and metabolism (Albumin[7-9], GLUT1[10,11], TKLP1[12,13]); **3**. biomarkers associated with tumor suppressive potential (FOXO1[14,15], FOX03[15,16], p53[17], PTEN[18], Wnt5a[19]); **4**. biomarkers associated with oncogenic potential (EPCAM[20], COX2[21,22], c-MET[23,24], Beta-Catenin[24,25], EZH2[26-30]); **5**. biomarkers associated with cell-cell adhesion, cellular and stromal structure (Beta-actin[31], Claudin-1[32], E-cadherin[33], EPCAM[20], Lamin A/C[34,35], CK19[36], NaKATPase[37], Fibronectin[38], Collagen IV[39]); **6**. biomarkers associated with post-translational modifications (PTM)[40] (p4EBP1, pMET, pERK1/2, pMAPKAPK2, p-p38MAPK, pEGFR, pGSK3a/b, pNDRG1, pS6); and **7**. biomarkers associated with cell types and their states (ALDH1[41,42], CD20[43,44], CD68[45], CD163[45], CD31[46], CD79[47], EZH2[26–30], CD3[48], CD8[49,50], PCK26[51], SMA[52,53], Fibronectin[38], NDRG1[54]).

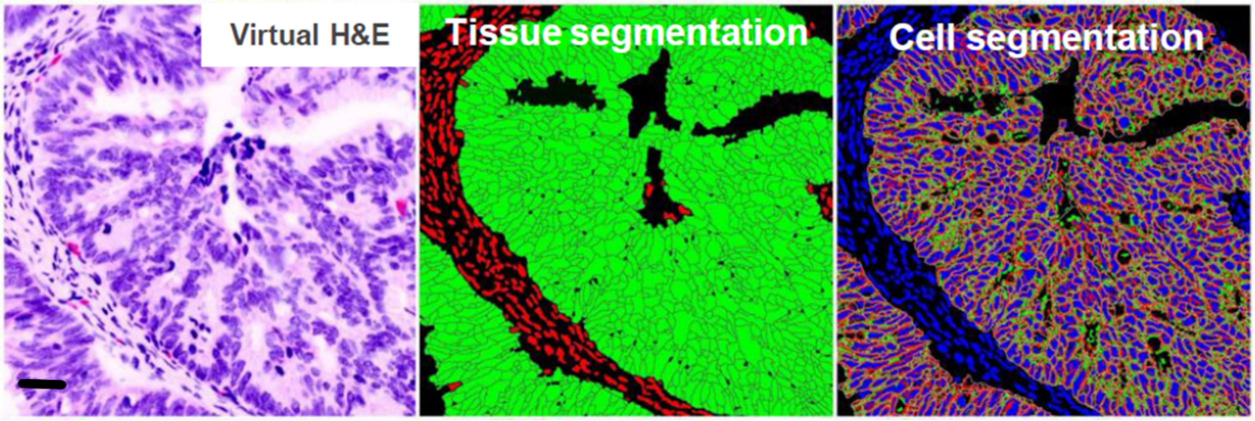

Supplementary Figure 3. Tissue and cell segmentation. TMA spot visualized here through a virtual Hematoxylin and Eosin image. (Scale bar = 50 µm.) Tissue segmentation is performed by using expression of E-cadherin, a highly epithelial cell-specific marker, to identify the epithelial spatial domain, shown in green. The remaining region of the TMA spot is identified as the stromal spatial domain, shown in red. The epithelial-stromal spatial-domain runs all along the boundary between the epithelial (green) and stromal (red) spatial-domains and has a width of 100 µm. Individual cell segmentation in the epithelial spatial domain is performed using expression of $Na^{+}K^{+}$ATPase (cell membrane marker), ribosomal protein S6 (cytoplasmic marker) and DAPI (nuclear counterstain, shown in blue). The remaining cells are assigned to the stromal spatial domain.

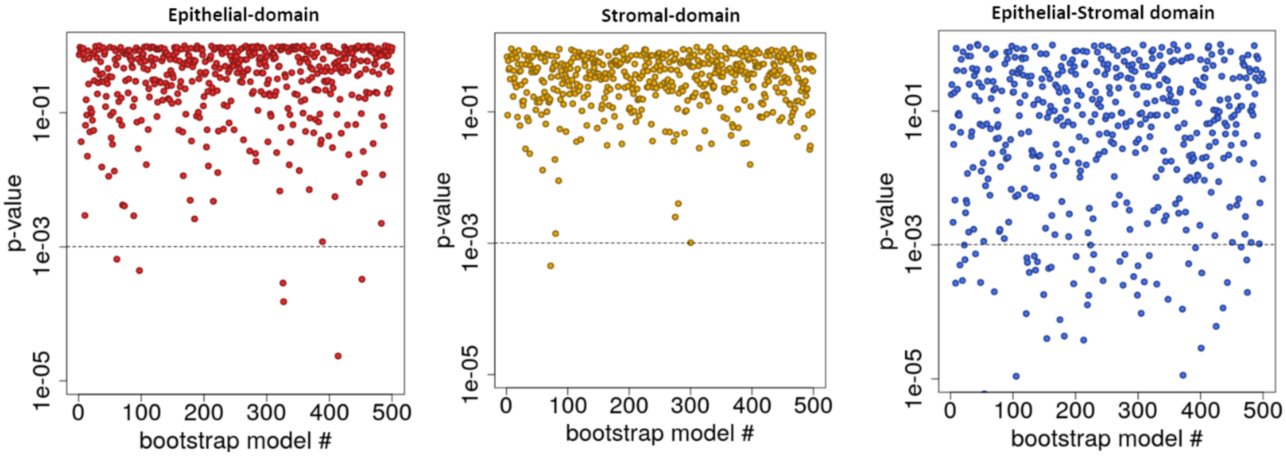

Supplementary Figure 4. Validity of the proportional hazard assumption in penalized Cox regression. The domain specific p-values (shown in log scale) measure the significance of the relationship between scaled Schoenfeld residuals and time to recurrence for all Cox models generated for the 500 bootstrap runs. A non-significant relationship between the two indicates the validity of the proportional hazard assumption for the overall Cox regression. It can be seen that for each of the three domains, the overall global test is not statistically significant at the 95% confidence level (indicated by the black dashed line) for the 500 bootstrap runs, demonstrating that the proportional hazard assumption is consistently valid. The p-values were computed using the cox.zph function in the survival R package. The use of different colors to render the p-values for the three spatial domains is exclusively to ensure better visual contrast.

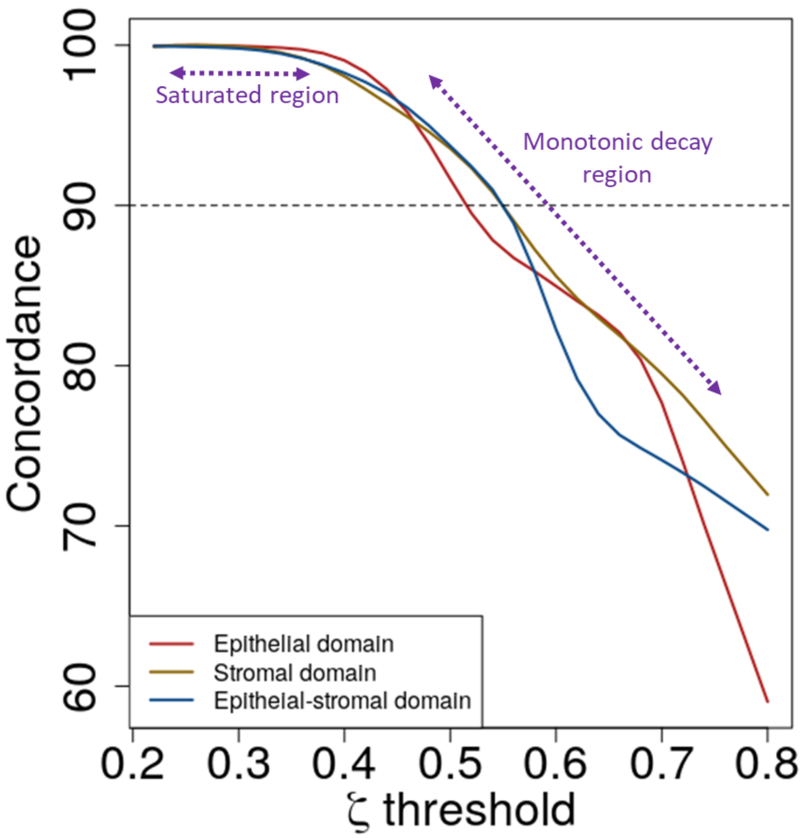

Supplementary Figure 5. Rationale for choosing 90% concordance rate. Plot of concordance of the penalized Cox regression model as a function of the threshold function that identifies the biomarker features most consistently selected by L1 penalization at the concordance level corresponding to the threshold. The larger the threshold the more stringent the consistency requirement on feature selection, and smaller the number of selected features. As shown in the plot, for low threshold values, the concordance value is saturated, and therefore, in this region injective correspondence between threshold value and concordance does not exist. In the monotonic decay region such a correspondence can be identified. The 90% concordance level, indicated by the black dashed line, identifies such a correspondence for all three spatial domains without compromising performance.

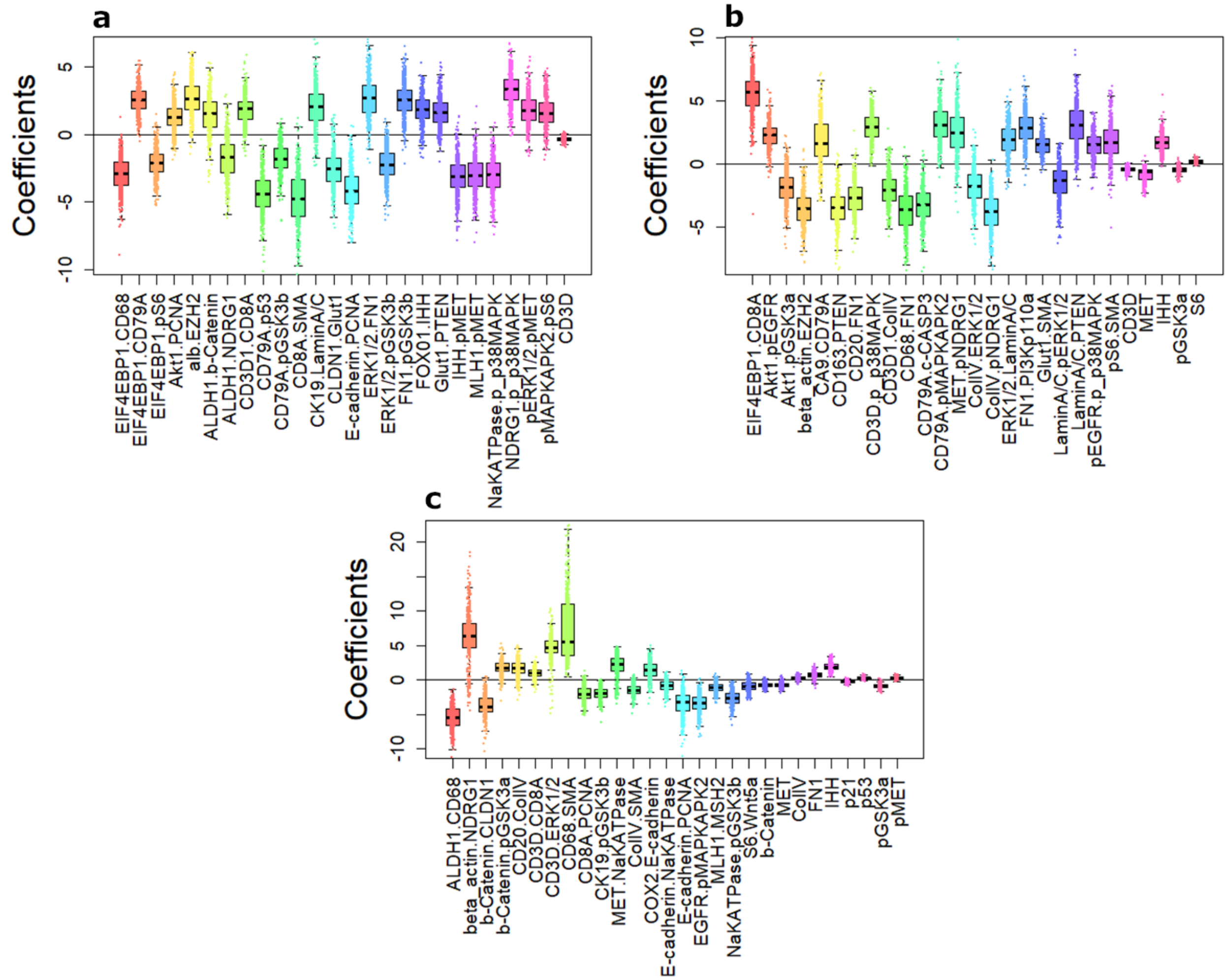

Supplementary Figure 6. Domain specific and recurrence-guided SpAn coefficients. Boxplots for coefficients that control the contribution of the selected features (obtained using L1-penalty) to each of the recurrence-guided and domain-specific penalized Cox regression under L2 regularization. The coefficients were computed for all 500 bootstrap runs and the boxplots capture the spread of values. The black sold line indicates zero coefficient value. Individual boxplots are colored exclusively for better visualization contrast. It is worth noting that for most bootstraps the coefficients maintain their sign, which quantifies the nature of their contribution. A positive coefficient implies worse prognosis for increase in the corresponding feature value, while negative coefficient implies the converse. (Box plot center line: median value; box bounds: interquartile range (IQR); upper whisker: 3rd quartile + 1.5 IQR; lower whisker: 1st quartile – 1.5 IQR.)

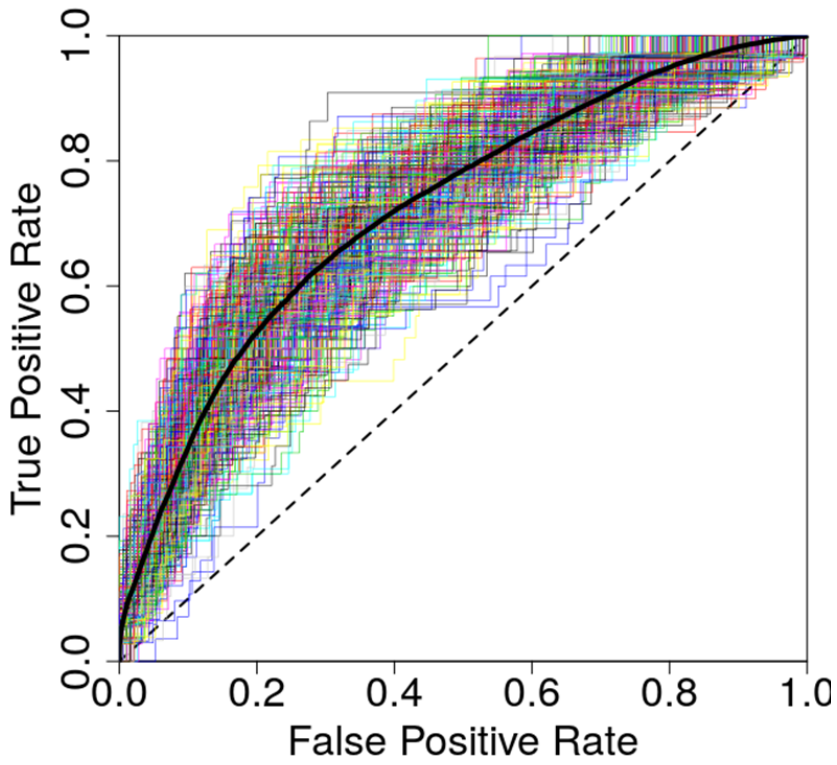

Supplementary Figure 7. Predicting 5-year CRC recurrence risk using only intensity-based features. SpAn ROC curves for predicting risk of 5-year CRC recurrence in patients with resected CRC primary tumor using only biomarker expressions. The plot shows ROC curves, rendered in different colors for improved visual contrast, for 500 bootstrap runs with independent training and validation sets. Area under the mean ROC curve, shown as a black solid curve, is 72% with a standard error of 0.2%. The black dashed 45-degree line indicates random guessing.

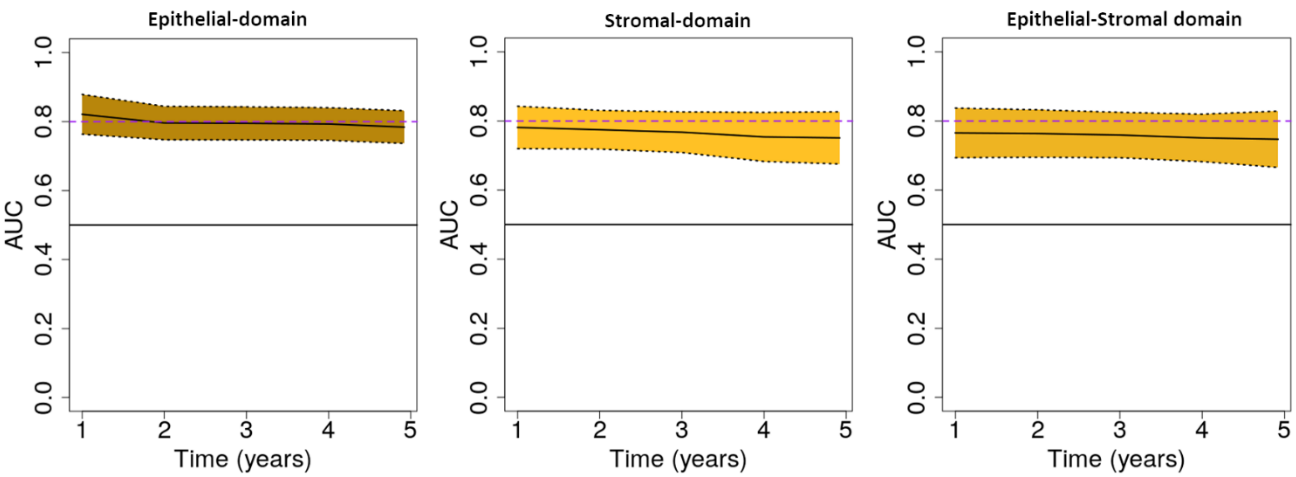

Supplementary Figure 8. Domain-specific temporal performance of SpAn. Temporal performance of SpAn for the three spatial-domains illustrated by the time-dependent AUC values plotted as a function of time in years. The 95% confidence interval computed using the 500 bootstraps for each of the three spatial-domains is also shown by the yellow shaded area around the mean time-dependent AUC values depicted by the black solid curve. The 0.8 and 0.5 AUC values are shown for reference by the purple-dashed and black-solid lines, respectively.

Supplementary Tables

Table S1. Biomarkers selection and groupings.

**Signaling Pathways**	PI3K/AKT/MTOR[1]	PI3Kp110a (Phosphatidylinositol-4,5-bisphosphate 3-kinase, catalytic subunit alpha),<br>4EBP1 (Eukaryotic translation initiation factor 4E-binding protein 1),<br>AKT1 (RAC-alpha serine/threonine-protein kinase),<br>EGFR (Epidermal growth factor receptor),<br>EZH2 (Enhancer of zeste homolog 2),<br>FOXO1 (Forkhead box protein O1),<br>FOXO3 Forkhead box protein O1),<br>p21 (Cyclin-dependent kinase inhibitor 1),<br>p53 (Tumor protein),<br>S6 (Ribosomal protein S6)
	RAS/RAF/MEK[2]	EGFR (Epidermal growth factor receptor),<br>ERK1/2 (Extracellular signal–regulated kinases),<br>EZH2 (Enhancer of zeste homolog 2),<br>S6 (Ribosomal protein S6)
	Mismatch Repair[3]	MLH1 (MutL homolog 1),<br>MSH2 (MutS protein homolog 2),<br>PCNA (Proliferating cell nuclear antigen),<br>p21 (Cyclin-dependent kinase inhibitor 1)
	Hedgehog signaling[4]	IHH (Indian hedgehog protein)
	Hypoxia-signaling[5]	CA9 (Carbonic anhydrase 9)
	Apoptosis[6]	c-CASP3 (Cleaved caspase-3)
**Extracellular transport and metabolism**		Alb[7-9] (Albumin),<br>GLUT1[10, 11] (Glucose transporter 1),<br>TKLP1[12, 13] (Transketolase-like protein 1)
**Tumor suppressive**		FOXO1[14, 15] (Forkhead box protein O1),<br>FOX03[15, 16] (Forkhead box protein O1),<br>p53[17] (tumor protein),<br>PTEN[18] (Phosphatase and tensin homolog),<br>Wnt5a[19] (Ligand for members of the frizzled family of seven transmembrane receptors)
**Oncogenic**		EpCAM[20] (Epithelial Cell Adhesion Molecule),<br>COX2[21, 22] (Cyclooxygenase-2),<br>c-MET[23, 24] (Tyrosine-protein kinase Met; also known as hepatocyte growth factor receptor),<br>β-Catenin[24, 25] (Catenin beta-1),<br>EZH2[26-30] (Enhancer of zeste homolog 2)
**Adhesion and structure**		Beta-actin[31] (one of six actin isoforms),<br>Claudin-1[32] (Transmembrane tight junction protein),<br>E-cadherin[33] (Epithelial calcium-dependent adhesion molecule),<br>EpCAM[20] (Epithelial cell adhesion molecule),

	Lamin A/C[34, 35] (nuclear lamina protein),<br>CK19[36] (Keratin, type I cytoskeletal 19),<br>NaKATPase[37] (Sodium–potassium pump; transmembrane ATPase enzyme)<br>Fibronectin[38] (Extracellular matrix glycoprotein),<br>Collagen IV[39] (Structural component of glomerular basement membranes)
**Post-translational modifications (PTM)**	Phosphorylated proteins[40] (p4EBP1, pMET, pERK1/2, pMAPKAPK2, p-p38MAPK, pEGFR, pGSK3a/b, pNDRG1, pS6)
**Cell types and states**	ALDH1[41, 42] (Aldehyde dehydrogenase 1 family, member A1),<br>CD20[43, 44] (B-cell differentiation antigen)<br>CD68[45] (Transmembrane glycoprotein macrophage marker),<br>CD163[45] (Scavenger receptor cysteine-rich type 1 protein M130; macrophage marker),<br>CD31[46] (Platelet endothelial cell adhesion molecule),<br>CD79[47] (B-cell antigen receptor complex-associated protein alpha chain),<br>EZH2[26–30] (Enhancer of zeste homolog 2),<br>CD3[48] (T-cell surface glycoprotein CD3 delta chain),<br>CD8[49, 50] (T-cell surface glycoprotein CD8 alpha chain),<br>PCK26[51] (Pan cytokeratin),<br>α-SMA[52, 53] (alpha smooth muscle actin),<br>Fibronectin[38] (Extracellular matrix glycoprotein),<br>NDRG1[54] (N-myc downstream-regulated gene 1)

Supplementary Table 2. Patient Cohort and Clinical Properties.

	All	StageI	StageII	StageIII
Patients	432	175	190	67
Age (median years)	75	67	73	70
Age (std)	6.7	11.7	11.4	13.4
Gender (Male)	220	88	101	31
Gender (% Male)	50.9	50.3	53.2	46.3
No. of Recurrences	65	10	30	25
Recurrence (%)	100	15.4	46.2	38.5
Recurrence Time (median days)	1651	1899	1574	1098
Survival Time (median days)	1695.5	1899	1658	1280

Supplementary Table 3. List of spatial-domain features (biomarkers and their correlations) selected by SpAn.

Epithelial.spatial.domain	Stromal.spatial.domain	Epithelial.Stromal.spatial.domain
4EBP1.CD68	4EBP1.CD8	ALDH1.CD68
4EBP1.CD79	Akt1.pEGFR	beta-actin.NDRG1
4EBP1.pS6	Akt1.pGSK3a	BetaCatenin.Claudin1
Akt1.PCNA	beta-actin.EZH2	BetaCatenin.pGSK3a
Albumin.EZH2	CA9.CD79	CD20.ColIV
ALDH1.BetaCatenin	CD163.PTEN	CD3.CD8
ALDH1.NDRG1	CD20.Fibronectin	CD3.ERK
CD31.CD8	CD3.p_p38MAPK	CD68.SMA
CD79.p53	CD31.ColIV	CD8.PCNA
CD79.pGSK3b	CD68.Fibronectin	CK19.pGSK3b
CD8.SMA	CD79.c-CASP3	c-MET.NaKATPase
CK19.LaminA/C	CD79.pMAPKAPK2	ColIV.SMA
Claudin1.Glut1	c-MET.pNDRG1	COX2.E-cadherin
E-cadherin.PCNA	ColIV.ERK	E-cadherin.NaKATPase
ERK.Fibronectin	ColIV.pNDRG1	E-cadherin.PCNA
ERK.pGSK3b	ERK.LaminA/C	EGFR.pMAPKAPK2
Fibronectin.pGSK3b	Fibronectin.PI3Kp110a	MLH1.MSH2
FOXO1.IHH	Glut1.SMA	NaKATPase.pGSK3b
Glut1.PTEN	LaminA/C.pERK	S6.Wnt5a
IHH.pMET	LaminA/C.PTEN	BetaCatenin
MLH1.pMET	pEGFR.p_p38MAPK	c-MET
NaKATPase.p_p38MAPK	pS6.SMA	ColIV
NDRG1.p_p38MAPK	CD3	Fibronectin
pERK.pMET	c-MET	IHH
pMAPKAPK2.pS6	IHH	p21
CD3	pGSK3a	p53
	S6	pGSK3a
		pMET

Supplementary Table 4. Comparing performance of SpAn with other prediction models. Statistical significance of pairwise performance comparison between SpAn and five different prediction models. The significance was estimated using Dunn's pairwise multiple comparison post-hoc analysis. The five prediction models include the clinical model, biomarker expression model (denoted by intensity), SpAn.null model (SpAn without spatial-domain context), biomarker expression + clinical model and SpAn + clinical model. All pairwise difference in performance are statistically significant at the 99% confidence interval except difference between 1. biomarker expression + clinical and null models, and 2. SpAn and SpAn + clinical models. Both have been highlighted by red solid rectangles.

Comparison	Z	P.unadj	P.adj
clinical - intensity	-3.169873	1.525058e-03	1.759683e-03
clinical - intensity+clinical	-20.289831	1.581377e-91	4.744131e-91
intensity - intensity+clinical	-17.119959	1.053504e-65	1.580256e-65
clinical - SpAn	-36.251476	9.418803e-288	7.064102e-287
intensity - SpAn	-33.081604	5.465671e-240	2.049627e-239
intensity+clinical - SpAn	-15.961645	2.364160e-57	3.223855e-57
clinical - SpAn.null	-18.593507	3.626737e-77	7.771579e-77
intensity - SpAn.null	-15.423634	1.135319e-53	1.419149e-53
intensity+clinical - SpAn.null	1.696324	8.982445e-02	8.982445e-02
SpAn - SpAn.null	17.657970	8.836122e-70	1.472687e-69
clinical - SpAn+clinical	-38.020964	0.000000e+00	0.000000e+00
intensity - SpAn+clinical	-34.851092	4.098566e-266	2.049283e-265
intensity+clinical - SpAn+clinical	-17.731133	2.411245e-70	4.521085e-70
SpAn - SpAn+clinical	-1.769488	7.681247e-02	8.229907e-02
SpAn.null - SpAn+clinical	-19.427458	4.522112e-84	1.130528e-83

Supplementary Table 5: List of antibodies (=63) and their imaging cycles. AF = autofluorescence; Biomarkers in blue rectangles (=8) were not used after applying quality control measures.

Imaging Round	1	2	3	4	5	6	7	8
Cy3 channel	AF	pERK1/2	β-Catenin	pS6	Vimentin	AF	xCT	GLUT1
Vendor		Cell Signaling Technology	Sigma	Cell Signaling Technology	Sigma		Epitomics	Millipore
Catalogue #		4370	C7738	4858	C9080		N/A	07-1401
Clone		20G11	15B8	D57.2.2E	V9		custom polyclonal	polyclonal
Staining Conc (ug/ml)		10	7.5	5	11		20	5
Cy5 channel	AF	CD31	S6	Fibronectin	Beta-actin	AF	PCK26	NaKATPases
Vendor		Cell Signaling Technology	Cell Signaling Technology	Epitomics	Cell Signaling Technology		Sigma	Epitomics
Catalogue #		3528	2217	1573	4970		C1801	2047-1
Clone		89C2	5G10	F1	13E5		PCK26	EP1845Y
Staining Conc (ug/ml)		5	5	10	10		2.5	5
Cy2 channel	AF	Empty	Empty	Empty	Empty	AF	HER2	α-SMA
Vendor		X	X	X	X		Cell Signaling Technology	Sigma
Catalogue #		X	X	X	X		4290	C6198
Clone		X	X	X	X		D8F12	1A4
Staining Conc (ug/ml)		X	X	X	X		10	5

Imaging Round	9	10	11	12	13	14	15	16
Cy3 channel	Collagen IV	CK19	AF	EGFR	Wnt5a	FOXO3	E-Cadherin	Lamin A/C
Vendor	Millipore	eBioscience		Cell Signaling Technology	Abcam	Epitomics	Cell Signaling Technology	Epitomics
Catalogue #	MAB3326	14-9898		4267	ab86720	2071-1	3195	2966-1
Clone	IV-4H12	BA17		D38B1	34D10	EP1949Y	24E10	EPR4100
Staining Conc (ug/ml)	5	10		10	10	15	5	20
Cy5 channel	Alb	Empty	AF	p4EBP1	pNDRG1	MLH1	pGSK3a	pGSK3b
Vendor	Sigma			Cell Signaling Technology	Cell Signaling Technology	Epitomics	Cell Signaling Technology	Epitomics
Catalogue #	a6684			2855	5482	2786-1	9316	2435
Clone	HAS-11			236B4	D98G11	EPR3894	36E9	EPR2286Y
Staining Conc (ug/ml)	10			2.5	10	10	10	10
Cy2 channel	Empty	Empty	AF	Empty	Empty	Empty	Empty	Empty
Vendor	X	X		X	X	X	X	X
Catalogue #	X	X		X	X	X	X	X
Clone	X	X		X	X	X	X	X
Staining Conc (ug/ml)	X	X		X	X	X	X	X

Imaging Round	17	18	19	20	21	22	23	24
Cy3 channel	EZH2	Cytokeratin15	Claudin-1	AF	CD44v6	IHH	NDRG1	TKLP1
Vendor	Cell Signaling Technology	Sigma	Sigma		eBioscience	Epitomics	Epitomics	AbD Serotec
Catalogue #	5246	HPA023910	wh0009076m1		BMS116	1910-1	5326-1	MCA5455Z
Clone	D2C9	polyclonal	1C5-D9		VFF-7	EP1192Y	EPR5592	1C10
Staining Conc (ug/ml)	20	5	10		5	20	5	10
Cy5 channel	ALDH1	p21	Empty	AF	CD20	pEGFR	CD68	CD8
Vendor	BD Transduction Laboratories	Cell Signaling Technology	X		Epitomics	Cell Signaling Technology	Thermo Scientific	Dako
Catalogue #	6111195	2947	X		1632-1	4407	MS-397	M7103
Clone	clone 11	12D1	X		EP459Y	53A5	KP1	DK25
Staining Conc (ug/ml)	10	5	X		10	10	5	2.5
Cy2 channel	Empty	Empty	Empty	AF	Empty	Empty	Empty	Empty
Vendor	X	X	X		X	X	X	X
Catalogue #	X	X	X		X	X	X	X
Clone	X	X	X		X	X	X	X
Staining Conc (ug/ml)	X	X	X		X	X	X	X

Imaging Round	25	26	27	28	29	30	31	32
Cy3 channel	CD79	p-MET	β-Tubulin	AKT1	CD79	AF	ERK1/2	EpCAM
Vendor	Dako	Epitomics	Sigma	Cell Signaling Technology	Dako		Cell Signaling Technology	Epitomics
Catalogue #	M7050	2319-1	C4585	4691	M7050		4695	3668-1
Clone	HM57	EP2367Y	TUB 2.1	C67E7	HM57		137F5	EPR677
Staining Conc (ug/ml)	10	5	10	20	10		10	10
Cy5 channel	PTEN	pMAPKAPK2	FOXO1	CA9	c-CASP3	AF	p38MAPK	CD3
Vendor	Cell Signaling Technology	Cell Signaling Technology	Cell Signaling Technology	Thermo Scientific	Cell Signaling Technology		Cell Signaling Technology	Dako
Catalogue #	9188	3007	2880	PA1-16592	9664		4511	M7254
Clone	D4.3	27B7	C29H4	polyclonal	5A1E		D3F9	F7.2.38
Staining Conc (ug/ml)	10	5	10	10	5		10	5
Cy2 channel	Empty	Empty	Empty	Empty	Empty	AF	Empty	Empty
Vendor	X	X	X	X	X		X	X
Catalogue #	X	X	X	X	X		X	X
Clone	X	X	X	X	X		X	X
Staining Conc (ug/ml)	X	X	X	X	X		X	X

Imaging Round	33	34	35	36	37	38
Cy3 channel	Empty	AF	CD163	PI3Kp110a	4EBP1	PCNA
Vendor	X		Leica	Cell Signaling Technology	Cell Signaling Technology	Cell Signaling Technology
Catalogue #	X		10D6	4249	9644	2586
Clone	X		NCL-CD163	C73F8	53H11	PC10
Staining Conc (ug/ml)	X		5	10	10	10
Cy5 channel	MSH2	AF	c-MET	Cyclin B1	COX2	p53
Vendor	Cell Signaling Technology		Epitomics	Epitomics	Invitrogen	Dako
Catalogue #	2017		S1354	1495	35-8200	M7001
Clone	D24B5		polyclonal	Y106	COX 229	DO-7
Staining Conc (ug/ml)	10		10	10	20	1
Cy2 channel	Empty	AF	Empty	Empty	Empty	Empty
Vendor	X		X	X	X	X
Catalogue #	X		X	X	X	X
Clone	X		X	X	X	X
Staining Conc (ug/ml)	X		X	X	X	X

Supplementary Table 6. Stratified sampling example of patients in whom CRC recurred in the first five years.

	< 1 year	1-2 years	2-3 years	3-4 years	4-5 years	> 5 years
High-risk CRC patients in Training set	9	9	4	5	5	2
High-risk CRC patients in Testing set	9	9	4	4	3	2

Supplementary References

1. Francipane, M. G. & Lagasse, E. mTOR pathway in colorectal cancer: an update. *Oncotarget* **5**, 49–66 (2014).

2. Burotto, M., Chiou, V. L., Lee, J.-M. & Kohn, E. C. The MAPK pathway across different malignancies: a new perspective. *Cancer* **120**, 3446–3456 (2014).

3. Li, G.-M. Mechanisms and functions of DNA mismatch repair. *Cell Res.* **18**, 85–98 (2008).

4. Wu, C., Zhu, X., Liu, W., Ruan, T. & Tao, K. Hedgehog signaling pathway in colorectal cancer: function, mechanism, and therapy. *Onco. Targets. Ther.* **10**, 3249–3259 (2017).

5. Greenhough, A. *et al.* Cancer cell adaptation to hypoxia involves a HIF-GPRC5A-YAP axis. *EMBO Mol. Med.* **10**, e8699 (2018).

6. Watson, A. J. M. Apoptosis and colorectal cancer. *Gut* **53**, 1701 LP – 1709 (2004).

7. González-Trejo, S. *et al.* Baseline serum albumin and other common clinical markers are prognostic factors in colorectal carcinoma: A retrospective cohort study. *Medicine (Baltimore).* **96**, (2017).

8. FUJIKAWA, H. *et al.* Prognostic Impact of Preoperative Albumin–to–Globulin Ratio in Patients with Colon Cancer Undergoing Surgery with Curative Intent. *Anticancer Res.* **37**, 1335–1342 (2017).

9. Huang, H. *et al.* Validation of Prognosis Value of Cumulative Prognostic Scores Based on Serum High-Density Lipoprotein Cholesterol and Albumin Levels in Patients with Colorectal Cancer. *J. Cancer* **10**, 35–42 (2019).

10. Feng, W. *et al.* Role of glucose metabolism related gene GLUT1 in the occurrence and prognosis of colorectal cancer. *Oncotarget* **8**, 56850–56857 (2017).

11. Shen, Y.-M., Arbman, G., Olsson, B. & Sun, X.-F. Overexpression of GLUT1 in Colorectal Cancer is Independently Associated with Poor Prognosis. *Int. J. Biol. Markers* **26**, 166–172 (2011).

12. Ahopelto, K., Böckelman, C., Hagström, J., Koskensalo, S. & Haglund, C. Transketolase-like protein 1 expression predicts poor prognosis in colorectal cancer. *Cancer Biol. Ther.* **17**, 163–168 (2016).

13. Langbein, S. *et al.* Expression of transketolase TKTL1 predicts colon and urothelial cancer patient survival: Warburg effect reinterpreted. *Br. J. Cancer* **94**, 578–585 (2006).

14. Pan, S. *et al.* Decreased expression of ARHGAP15 promotes the development of colorectal cancer through PTEN/AKT/FOXO1 axis. *Cell Death Dis.* **9**, 673 (2018).

15. Farhan, M. *et al.* FOXO Signaling Pathways as Therapeutic Targets in Cancer. *Int. J. Biol. Sci.* **13**, 815–827 (2017).

16. Bullock, M. D. *et al.* FOXO3 expression during colorectal cancer progression: biomarker potential reflects a tumour suppressor role. *Br. J. Cancer* **109**, 387–394 (2013).

17. Li, X.-L., Zhou, J., Chen, Z.-R. & Chng, W.-J. P53 mutations in colorectal cancer - molecular pathogenesis and pharmacological reactivation. *World J. Gastroenterol.* **21**, 84–93 (2015).

18. Molinari, F. & Frattini, M. Functions and Regulation of the PTEN Gene in Colorectal Cancer. *Front. Oncol.* **3**, 326 (2014).

19. Ying, J. *et al.* WNT5A Exhibits Tumor-Suppressive Activity through Antagonizing the Wnt/β-Catenin Signaling, and Is Frequently Methylated in Colorectal Cancer. *Clin. Cancer Res.* **14**, 55 LP – 61 (2008).

20. Munz, M., Baeuerle, P. A. & Gires, O. The Emerging Role of EpCAM in Cancer and Stem Cell Signaling. *Cancer Res.* **69**, 5627 LP – 5629 (2009).

21. Greenhough, A. *et al.* The COX-2/PGE 2 pathway: key roles in the hallmarks of cancer and adaptation to the tumour microenvironment . *Carcinogenesis* **30**, 377–386 (2009).

22. Wang, D. & DuBois, R. N. The role of COX-2 in intestinal inflammation and colorectal cancer. *Oncogene* **29**, 781–788 (2010).

23. Lee, S. J. *et al.* c-MET Overexpression in Colorectal Cancer: A Poor Prognostic Factor for Survival. *Clin. Colorectal Cancer* **17**, 165–169 (2018).

24. Rasola, A. *et al.* A positive feedback loop between hepatocyte growth factor receptor and β-catenin sustains colorectal cancer cell invasive growth. *Oncogene* **26**, 1078–1087 (2007).

25. Shang, S., Hua, F. & Hu, Z.-W. The regulation of β-catenin activity and function in cancer: therapeutic opportunities. *Oncotarget* **8**, 33972–33989 (2017).

26. Gan, L. *et al.* Epigenetic regulation of cancer progression by EZH2: from biological insights to therapeutic potential. *Biomark. Res.* **6**, 10 (2018).

27. Vilorio-Marqués, L. *et al.* The role of EZH2 in overall survival of colorectal cancer: a meta-analysis. *Sci. Rep.* **7**, 13806 (2017).

28. Karantanos, T., Chistofides, A., Barhdan, K., Li, L. & Boussiotis, V. A. Regulation of T Cell Differentiation and Function by EZH2. *Front. Immunol.* **7**, 172 (2016).

29. Goswami, S. *et al.* Modulation of EZH2 expression in T cells improves efficacy of anti–CTLA-4 therapy. *J. Clin. Invest.* **128**, 3813–3818 (2018).

30. DuPage, M. *et al.* The chromatin-modifying enzyme Ezh2 is critical for the maintenance of regulatory T cell identity after activation. *Immunity* **42**, 227–238 (2015).

31. Simiczyjew, A., Mazur, A. J., Popow-Woźniak, A., Malicka-Błaszkiewicz, M. & Nowak, D. Effect of overexpression of β- and γ-actin isoforms on actin cytoskeleton organization and migration of human colon cancer cells. *Histochem. Cell Biol.* **142**, 307–322 (2014).

32. Dhawan, P. *et al.* Claudin-1 regulates cellular transformation and metastatic behavior in colon cancer. *J. Clin. Invest.* **115**, 1765–1776 (2005).

33. Kim, S. A. *et al.* Loss of CDH1 (E-cadherin) expression is associated with infiltrative tumour growth and lymph node metastasis. *Br. J. Cancer* **114**, 199–206 (2016).

34. Willis, N. D. *et al.* Lamin A/C is a risk biomarker in colorectal cancer. *PLoS One* **3**, e2988–e2988 (2008).

35. Belt, E. J. T. *et al.* Loss of lamin A/C expression in stage II and III colon cancer is associated with disease recurrence. *Eur. J. Cancer* **47**, 1837–1845 (2011).

36. Jain, R., Fischer, S., Serra, S. & Chetty, R. The Use of Cytokeratin 19 (CK19) Immunohistochemistry in Lesions of the Pancreas, Gastrointestinal Tract, and Liver. *Appl. Immunohistochem. Mol. Morphol.* **18**, (2010).

37. Clausen, M. V, Hilbers, F. & Poulsen, H. The Structure and Function of the Na,K-ATPase Isoforms in Health and Disease. *Front. Physiol.* **8**, 371 (2017).

38. Wang, J. P. & Hielscher, A. Fibronectin: How Its Aberrant Expression in Tumors May Improve Therapeutic Targeting. *J. Cancer* **8**, 674–682 (2017).

39. Tanjore, H. & Kalluri, R. The role of type IV collagen and basement membranes in cancer progression and metastasis. *Am. J. Pathol.* **168**, 715–717 (2006).

40. Ardito, F., Giuliani, M., Perrone, D., Troiano, G. & Lo Muzio, L. The crucial role of protein phosphorylation in cell signaling and its use as targeted therapy (Review). *Int. J. Mol. Med.* **40**, 271–280 (2017).

41. Hessman, C. J., Bubbers, E. J., Billingsley, K. G., Herzig, D. O. & Wong, M. H. Loss of expression of the cancer stem cell marker aldehyde dehydrogenase 1 correlates with advanced-stage colorectal cancer. *Am. J. Surg.* **203**, 649–653 (2012).

42. Douville, J., Beaulieu, R. & Balicki, D. ALDH1 as a Functional Marker of Cancer Stem and Progenitor Cells. *Stem Cells Dev.* **18**, 17–26 (2008).

43. Nelson, B. H. CD20⁺ B Cells: The Other Tumor-Infiltrating Lymphocytes. *J. Immunol.* **185**, 4977 LP – 4982 (2010).

44. Berntsson, J., Nodin, B., Eberhard, J., Micke, P. & Jirström, K. Prognostic impact of tumour-infiltrating B cells and plasma cells in colorectal cancer. *Int. J. Cancer* **139**, 1129–1139 (2016).

45. Pinto, M. L. *et al.* The Two Faces of Tumor-Associated Macrophages and Their Clinical Significance in Colorectal Cancer. *Front. Immunol.* **10**, 1875 (2019).

46. Lu, J. *et al.* Endothelial cells promote the colorectal cancer stem cell phenotype through a soluble form of Jagged-1. *Cancer Cell* **23**, 171–185 (2013).

47. Chu, P. G. & Arber, D. A. CD79: A Review. *Appl. Immunohistochem. Mol. Morphol.* **9**, (2001).

48. Cavalleri, T. *et al.* Combined Low Densities of FoxP3⁺ and CD3⁺ Tumor-Infiltrating Lymphocytes Identify Stage II Colorectal Cancer at High Risk of Progression. *Cancer Immunol. Res.* **7**, 751 LP – 758 (2019).

49. Ziai, J. *et al.* CD8+ T cell infiltration in breast and colon cancer: A histologic and statistical analysis. *PLoS One* **13**, e0190158–e0190158 (2018).

50. Zhang, S. *et al.* CCL5-deficiency enhances intratumoral infiltration of CD8+ T cells in colorectal cancer. *Cell Death Dis.* **9**, 766 (2018).

51. Schweizer, J. *et al.* New consensus nomenclature for mammalian keratins. *J. Cell Biol.* **174**, 169–174 (2006).

52. Liu, T., Zhou, L., Li, D., Andl, T. & Zhang, Y. Cancer-Associated Fibroblasts Build and Secure the Tumor Microenvironment . *Frontiers in Cell and Developmental Biology* **7**, 60 (2019).

53. Nishishita, R. *et al.* Expression of cancer-associated fibroblast markers in advanced colorectal cancer. *Oncol. Lett.* **15**, 6195–6202 (2018).

54. Mi, L. *et al.* The metastatic suppressor NDRG1 inhibits EMT, migration and invasion through interaction and promotion of caveolin-1 ubiquitylation in human colorectal cancer cells. *Oncogene* **36**, 4323–4335 (2017).
