# Supplementary material for: Spatial domain analysis predicts risk of colorectal cancer recurrence and infers associated tumor microenvironment networks
Source: Nat Commun. 2020 Jul 14;11:3515. doi: 10.1038/s41467-020-17083-x (PMC7360741; doi:10.1038/s41467-020-17083-x)
Supplement: Supplementary file 2 — Reporting Summary [file 41467_2020_17083_MOESM2_ESM.pdf]

nature research

Corresponding author(s): S. Chakra Chennubhotla

Last updated by author(s): Jul 15, 2019

Reporting Summary

Nature Research wishes to improve the reproducibility of the work that we publish. This form provides structure for consistency and transparency in reporting. For further information on Nature Research policies, see Authors & Referees and the Editorial Policy Checklist.

Statistics

For all statistical analyses, confirm that the following items are present in the figure legend, table legend, main text, or Methods section.

n/a	Confirmed	
☐	☒	The exact sample size (n) for each experimental group/condition, given as a discrete number and unit of measurement
☐	☒	A statement on whether measurements were taken from distinct samples or whether the same sample was measured repeatedly
☐	☒	The statistical test(s) used AND whether they are one- or two-sided<br>*Only common tests should be described solely by name; describe more complex techniques in the Methods section.*
☐	☒	A description of all covariates tested
☐	☒	A description of any assumptions or corrections, such as tests of normality and adjustment for multiple comparisons
☐	☒	A full description of the statistical parameters including central tendency (e.g. means) or other basic estimates (e.g. regression coefficient) AND variation (e.g. standard deviation) or associated estimates of uncertainty (e.g. confidence intervals)
☐	☒	For null hypothesis testing, the test statistic (e.g. F, t, r) with confidence intervals, effect sizes, degrees of freedom and P value noted<br>*Give P values as exact values whenever suitable.*
☒	☐	For Bayesian analysis, information on the choice of priors and Markov chain Monte Carlo settings
☒	☐	For hierarchical and complex designs, identification of the appropriate level for tests and full reporting of outcomes
☒	☐	Estimates of effect sizes (e.g. Cohen's d, Pearson's r), indicating how they were calculated

Our web collection on statistics for biologists contains articles on many of the points above.

Software and code

Policy information about availability of computer code

Data collection	CRC data used in this paper was previously published in Gerdes, M. J. et al. Highly multiplexed single-cell analysis of formalin-fixed, paraffin embedded cancer tissue. Proc. Natl. Acad. Sci. U. S. A. 110, 11982–11987 (2013).
Data analysis	The custom algorithms were coded in R.

For manuscripts utilizing custom algorithms or software that are central to the research but not yet described in published literature, software must be made available to editors/reviewers. We strongly encourage code deposition in a community repository (e.g. GitHub). See the Nature Research guidelines for submitting code & software for further information.

Data

Policy information about availability of data

All manuscripts must include a data availability statement. This statement should provide the following information, where applicable:

- Accession codes, unique identifiers, or web links for publicly available datasets
- A list of figures that have associated raw data
- A description of any restrictions on data availability

Data used in the computation and generation of results and figures in this manuscript will be made available.

Field-specific reporting

Please select the one below that is the best fit for your research. If you are not sure, read the appropriate sections before making your selection.

☒ Life sciences ☐ Behavioural & social sciences ☐ Ecological, evolutionary & environmental sciences

October 2018

Life sciences study design

All studies must disclose on these points even when the disclosure is negative.

Sample size	Sample size is explicitly stated along with number of features measured per sample
Data exclusions	Quality control measures applied include cell filtering, spot exclusion, log2 transformation and slide to slide normalization. More details in the quality checks and data normalization subsection of the Methods section.
Replication	Models were trained over 500 bootstrap runs with each condition to ensure the stability of the training procedure. Replication was successful for all conditions for which test results were reported.
Randomization	Patients were randomly divided into independent training and testing datasets using 500 stratified bootstraps.
Blinding	Not necessary for this study as this study uses digitized immunofluorescence labeled tissue slides

Reporting for specific materials, systems and methods

We require information from authors about some types of materials, experimental systems and methods used in many studies. Here, indicate whether each material, system or method listed is relevant to your study. If you are not sure if a list item applies to your research, read the appropriate section before selecting a response.

Materials & experimental systems

n/a	Involved in the study	
☐	☒	Antibodies
☒	☐	Eukaryotic cell lines
☒	☐	Palaeontology
☒	☐	Animals and other organisms
☐	☒	Human research participants
☒	☐	Clinical data

Methods

n/a	Involved in the study	
☒	☐	ChIP-seq
☒	☐	Flow cytometry
☒	☐	MRI-based neuroimaging

Antibodies

Antibodies used	Antibody information detailed in Gerdes et al Highly multiplexed single-cell analysis of formalin-fixed, paraffin embedded cancer tissue. Proc. Natl. Acad. Sci. U. S. A. 110, 11982–11987 (2013).
Validation	Validation details in Gerdes et al Highly multiplexed single-cell analysis of formalin-fixed, paraffin embedded cancer tissue. Proc. Natl. Acad. Sci. U. S. A. 110, 11982–11987 (2013).

Human research participants

Policy information about studies involving human research participants

Population characteristics	As described in Gerdes et al Highly multiplexed single-cell analysis of formalin-fixed, paraffin embedded cancer tissue. Proc. Natl. Acad. Sci. U. S. A. 110, 11982–11987 (2013), colorectal cancer TMAs were provided by Clarient Inc. The colorectal cancer cohort was collected from the Clearview Cancer Institute of Huntsville Alabama from 1993 until 2002, in the form of paraffin-embedded specimens. See Supp Table S2 for more details.
Recruitment	No patient recruitment was performed. Hyperplexed images that were available for 432 chemo-naive patients in the pre-established collecting period in Gerdes et al Highly multiplexed single-cell analysis of formalin-fixed, paraffin embedded cancer tissue. Proc. Natl. Acad. Sci. U. S. A. 110, 11982–11987 (2013) paper were analyzed.
Ethics oversight	Clearview Cancer Institute of Huntsville Alabama

Note that full information on the approval of the study protocol must also be provided in the manuscript.
